# Large‐scale genetic screens identify BET‐1 as a cytoskeleton regulator promoting actin function and life span

**DOI:** 10.1111/acel.13742

**Published:** 2022-11-20

**Authors:** Gilberto Garcia, Raz Bar‐Ziv, Maxim Averbukh, Nirmalya Dasgupta, Naibedya Dutta, Hanlin Zhang, Wudi Fan, Darius Moaddeli, C. Kimberly Tsui, Toni Castro Torres, Athena Alcala, Erica A. Moehle, Sally Hoang, Ophir Shalem, Peter D. Adams, Max A. Thorwald, Ryo Higuchi‐Sanabria

**Affiliations:** ^1^ Leonard Davis School of Gerontology University of Southern California Los Angeles California USA; ^2^ Department of Molecular & Cellular Biology, Howard Hughes Medical Institute The University of California, Berkeley Berkeley California USA; ^3^ Aging, Cancer and Immuno‐oncology Program Sanford Burnham Prebys Medical Discovery Institute La Jolla California USA; ^4^ Department of Genetics, Perelman School of Medicine University of Pennsylvania Philadelphia Pennsylvania USA

**Keywords:** actin, aging, CRISPR

## Abstract

The actin cytoskeleton is a three‐dimensional scaffold of proteins that is a regulatory, energyconsuming network with dynamic properties to shape the structure and function of the cell. Proper actin function is required for many cellular pathways, including cell division, autophagy, chaperone function, endocytosis, and exocytosis. Deterioration of these processes manifests during aging and exposure to stress, which is in part due to the breakdown of the actin cytoskeleton. However, the regulatory mechanisms involved in preservation of cytoskeletal form and function are not well‐understood. Here, we performed a multipronged, cross‐organismal screen combining a whole‐genome CRISPR‐Cas9 screen in human fibroblasts with in vivo *Caenorhabditis elegans* synthetic lethality screening. We identified the bromodomain protein, BET‐1, as a key regulator of actin function and longevity. Overexpression of *bet‐1* preserves actin function at late age and promotes life span and healthspan in *C. elegans*. These beneficial effects are mediated through actin preservation by the transcriptional regulator function of BET‐1. Together, our discovery assigns a key role for BET‐1 in cytoskeletal health, highlighting regulatory cellular networks promoting cytoskeletal homeostasis.

## Graphical abstract

Using a cross‐species screening approach combining genome‐wide CRISPR‐Cas9 screening in human fibroblasts (1) and synthetic lethality screening in *Caenorhabditis elegans* (2), we identified BET‐1/BRD4 as a novel regulator of the actin cytoskeleton (3). BET‐1/BRD4 promotes actin filament stability in *C. elegans* and senescent cells, which has direct impact on organismal health, survival, and life span.

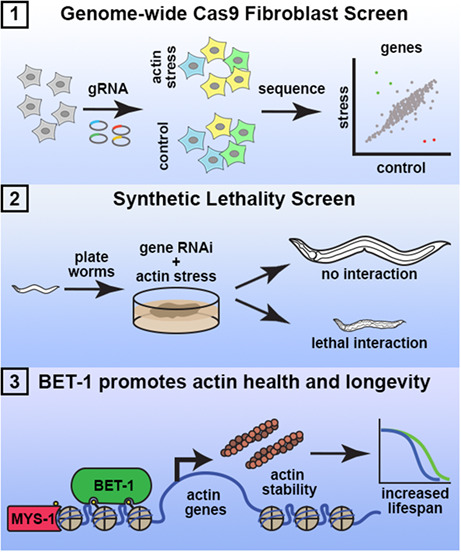

**Abbreviations**
ACSR: actin cytoskeletal stress response
EV: empty vector
GO: gene ontology
RNAi: ribonucleic acid interference

## 1 INTRODUCTION

The actin cytoskeleton is composed of a complex network of filaments held together by actin‐binding proteins. Historically, the actin cytoskeleton has been viewed as merely the structural framework of the cell, with its primary function being ascribed to cell division and the sorting and transport of cellular cargo. However, actin function is also required for many other cellular pathways, including autophagy, chaperone function, and transcriptional regulation (Balch et al., 2008; Blanpied et al., 2003; Caviston & Holzbaur, 2006; Higuchi‐Sanabria et al., 2014; McCray & Taylor, 2008). The functional significance of actin in cellular health and disease is highlighted by clinical data, showing that loss of actin function is observed in clinical manifestations, including neurodegeneration and muscle myopathies (Acsadi et al., 1991; Alim et al., 2002; Alim et al., 2004), and during aging (Baird et al., 2014; Higuchi‐Sanabria et al., 2018; Sing et al., 2021). Specifically, actin filaments show loss of stability and marked deterioration during aging in both single‐celled yeast (Sing et al., 2021) and multiple cell‐types of the multicellular nematode *Caenorhabditis elegans* (Higuchi‐Sanabria et al., 2018), whereas changes in β‐actin expression have been documented in mammals (Moshier et al., 1993). Here, we hypothesize that still unidentified pathways may promote actin function, which can be tailored to protect the cytoskeleton, and their function might be compromised with age, contributing to decline in cellular and organismal health.

One recently identified mechanism by which the cell protects its cytoskeleton during stress is through the heat‐shock response (HSR), mediated by the heat‐shock transcription factor, HSF‐1. HSF‐1 is activated under thermal stress and promotes protein homeostasis through the upregulation of chaperones and other genes related to protein quality control (Morley & Morimoto, 2004). Overexpression of *hsf‐1* is sufficient to confer an increase in thermal stress tolerance and life span in *C. elegans*, and alleviates the toxic effects associated with aging (Morley & Morimoto, 2004). Interestingly, overexpression of a truncated variant of *hsf‐1* lacking the capacity to upregulate heat‐shock chaperones was also sufficient to extend life span (Baird et al., 2014). Overexpression of this truncated form of *hsf‐1* upregulated genes involved in the maintenance of the actin cytoskeleton, including the troponin C/calmodulin homolog, *pat‐10*, which is both sufficient and necessary for HSF‐1‐mediated thermotolerance and longevity (Baird et al., 2014). However, many actin‐regulating genes are not bona fide targets of HSF‐1, suggesting the existence of other master regulators, which function independently or in parallel with HSF‐1 to modulate actin and protect the cytoskeleton. For example, the general regulation of actin is governed by key actin nucleation factors that fall into three major classes: (1) the Arp2/3 (actin‐related protein 2/3) complex that builds branched actin networks (Machesky et al., 1994; Machesky et al., 1999); (2) formins that build unbranched actin filaments (Pruyne et al., 2002); and (3) tandem‐monomer binding nucleators, which bring monomers together to form actin nucleation seeds (Quinlan et al., 2005; Sagot et al., 2002). All three types of actin nucleation factors were not found to be regulated by HSF‐1, and the beneficial effect of *hsf‐1* overexpression was actually independent of the tropomyosin, *lev‐11* (LEVamisole resistant) (Baird et al., 2014).

To identify previously unidentified regulators of actin function, we adopted a multipronged, cross‐organismal screening approach. Since actin is one of the most highly conserved proteins, both in terms of sequence and functional homology, we held the rationale that an evolutionary‐conserved perspective could provide a powerful method to identify key regulators of actin function. We first utilized a CRISPR/Cas9‐driven growth‐based genetic screen in human cells to identify genes required for survival under actin stress. Actin stress was applied by exposure to cytochalasin D, a drug that inhibits actin polymerization by binding to F‐actin filaments and preventing the addition of actin monomers (May et al., 1998). We then performed a secondary screen of the top ~500 candidate genes in the multicellular nematode, *C. elegans*, allowing us not only to explore its cellular contributions, but also to associate physiological manifestations in a multicellular organism. Our secondary screen consisted of a synthetic lethality screen to identify genes that when knocked down cause lethality in animals exposed to a sublethal knockdown of actin. These screens identified *bet‐1* (two bromodomain family protein), which promotes actin function, longevity, and healthspan in a multicellular eukaryote.

## 2 RESULTS

### 2.1 Cross‐species screening to identify novel regulators of Actin health

**FIGURE 1:**
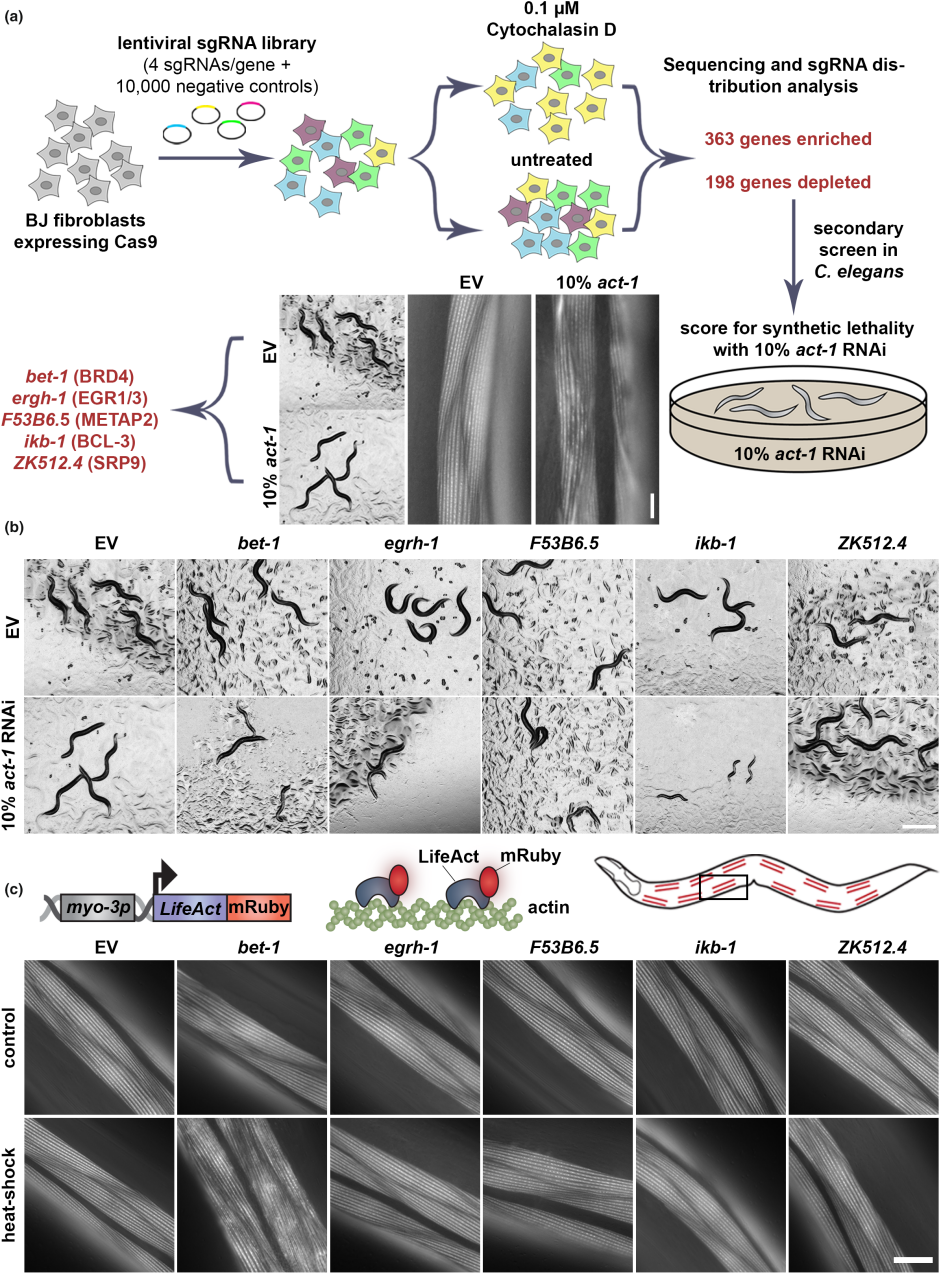
Multiplex screening reveals *bet‐1* is involved in regulation of actin. (a) Schematic of cross‐species screening. CRISPR‐Cas9 screening was performed in BJ fibroblasts with 0.1 μM cytochalasin D and top hits were screened in *Caenorhabditis elegans* with 90% candidate RNAi and 10% *act‐1* RNAi. (b) Images of N2 animals grown on 90% candidate RNAi mixed with either 10% empty vector (EV) or 10% *act‐1*. Hits were defined as those that exhibit an observable phenotype when combined with *act‐1* RNAi, but not with EV. Scale bar is 500 μm. (c) LifeAct::mRuby is expressed specifically in the body wall muscle cells with the *myo‐3* promoter. LifeAct::mRuby binds to F‐actin filaments to allow visualization of actin. Representative fluorescent images of body wall muscle actin are shown. Animals were grown on RNAi from hatch. Animals were heat‐shocked for 1 h at 37°C and immediately imaged. Images were captured on a Zeiss AxioObserver.Z1. Scale bar is 10 μm.

In our work to identify genes critical for actin function, we chose to inhibit actin polymerization using the chemical drug, cytochalasin D. Cytochalasin D prevents actin polymerization by binding to F‐actin filaments and blocking the addition of actin monomers (May et al., 1998). We screened for a concentration of cytochalasin D that only mildly affected growth rate (Figure S1a) and performed a CRISPR/Cas9‐driven reverse genetic screen in BJ fibroblasts, a karyotypically normal human fibroblast line. A whole‐genome CRISPR knockout screen was performed using the AVANA pooled sgRNA library (Schinzel et al., 2019; Shalem et al., 2014) in cells treated with 0.1 μM cytochalasin D (Figure 1a), which we confirmed does not impact cell survival (Figure S1b). We compared gene‐based depletion *p*‐values between control and treatment arms to curate a list of genes that were enriched or depleted in our cytochalasin‐treated cells (Tables S1 and S2, Figure S2a). Importantly, one of the most significantly depleted genes was the actin‐encoding gene, ACTB. Moreover, gene ontology (GO) analysis (Chen et al., 2013; Kuleshov et al., 2016; Xie et al., 2021) revealed actin cytoskeleton, the general cytoskeleton, and actin filaments as significantly enriched GO terms for the top depleted genes (Figure S2b, Table S3). These data provide confidence that our screen is revealing genes important for actin function. Interestingly, many mitochondrial genes were found amongst significantly enriched genes (Figure S2c, Table S4), though this is consistent with many findings that have previously revealed actin‐mitochondrial interactions (Fehrenbacher et al., 2004; Higuchi et al., 2013; Moehle et al., 2021; Tharp et al., 2021).

To follow up on the identified hits and link their contribution to cellular and organismal physiology, we opted for performing a cross‐species analysis in the nematode *C. elegans* with the rationale that genes important for surviving actin stress in two species must be critical regulators of actin function. Indeed, we found in a previous study that similar cross‐species screening approaches provided more biologically meaningful data than computational methods alone to identify candidate genes that impact life span (Moehle et al., 2021). Therefore, we searched the top ~500 enriched and depleted genes from our screen for evolutionary‐conserved orthologs in *C. elegans* (Kim et al., 2018) and identified a list of ~400 genes. Next, we performed a synthetic lethality screen for these candidate genes, searching for genes that when knocked down, caused a synthetic sick or synthetic lethal phenotype when combined with a sub‐lethal knockdown of actin (10% *act‐1* RNAi) (Figure 1a). Importantly, the selected RNAi sequence of *act‐1* shows overlap with all 5 isoforms of actin in *C. elegans* (*act‐1* through *act‐5*) and exhibits visible perturbations of actin function without affecting whole organismal physiology. Animals treated with 10% *act‐1* RNAi develop normally to adulthood but display notable perturbations of actin quality in muscle cells (Figure 1a). Therefore, we proceeded with our synthetic lethality screen by performing RNAi knockdown of candidate genes mixed with *act‐1* RNAi in a 9:1 ratio, respectively.

Our secondary screen revealed 5 potential candidate genes with varying phenotypes (Figure 1b). RNAi knockdown of *bet‐1*, *egrh‐1* (Early Growth factor Response factor Homolog), *F53B6.5*, and *ikb‐1* (I Kappa B homolog) resulted in delayed development when combined with 10% *act‐1* RNAi but exhibit no physiological phenotypes when knocked down alone. RNAi knockdown of *ZK512.4* did not exhibit a developmental defect but showed sterility (no eggs were visible on the plate at day 1 of adulthood) when combined with 10% *act‐1* RNAi, despite showing visible progeny formation when knocked down alone. *ZK512.4* is a predicted ortholog of human SRP9 (signal recognition particle), a critical part of protein targeting to the endoplasmic reticulum (ER; Mary et al., 2010), with no known association with the cytoskeleton, though studies have shown that actin can impact ER dynamics (Korobova et al., 2013; Poteryaev et al., 2005). *egrh‐1* is the ortholog of human EGR1 (early growth response 1), a major transcription factor that has been implicated in multiple diseases including cancer (Wang et al., 2021), neuropsychiatric disorders (Duclot & Kabbaj, 2017), and Alzheimer's disease (Qin et al., 2016). *F53B6.5* is a predicted ortholog of human methionyl aminopeptidase 2 (METAP2), which removes methionine residues from nascent polypeptide chains and are also implicated in cancer (Yin et al., 2012). *bet‐1* is the ortholog of human BRD4, a bromodomain protein involved in cell fate decisions in both *C. elegans* (Shibata et al., 2010) and mammals (Lee et al., 2017; Linares‐Saldana et al., 2021), and also implicated in cancer progression (Huang et al., 2016).

Next, we sought to determine which of our candidate genes directly impacted actin integrity and life span. To directly measure actin organization, we used animals expressing LifeAct::mRuby in the muscle. Here, the F‐actin binding protein, LifeAct is fused to a fluorescent molecule to allow robust visualization of actin integrity in muscle cells (Higuchi‐Sanabria et al., 2018). RNAi knockdown of *bet‐1* consistently resulted in decreased life span, whereas RNAi knockdown of *egrh‐1*, *F53B6.5*, and *ikb‐1* resulted in inconsistent changes to life span (Figure S3a, Tables S6 and S7). Moreover, a *bet‐1* mutant generated by introducing a premature stop codon at amino acid 17, consistently showed a significant decrease in life span (Figure S3b). Surprisingly, RNAi knockdown of any of the five candidate genes had no impact on actin integrity (Figure 1c, top). However, our previous work showed that RNAi knockdown of *hsf‐1*, which has dramatic effects on organismal health and life span (Hsu et al., 2003; Morley & Morimoto, 2004), also did not impact actin organization in the muscle in young, unstressed adults, and instead only exhibited measurable phenotypes during stress or aging (Higuchi‐Sanabria et al., 2018). Therefore, we applied an acute exposure to heat stress (1 h at 37°C), which has no measurable effect on actin organization in wild‐type animals. However, RNAi knockdown of *bet‐1*—and no other candidate genes—resulted in a dramatic loss of actin integrity and organization under acute heat stress (Figure 1c, bottom). Therefore, as a gene that directly impacts actin integrity and aging, we pursued *bet‐1* for follow‐up analysis.

### 2.2 *BET‐1* is a bonafide regulator of actin health during aging

**FIGURE 2:**
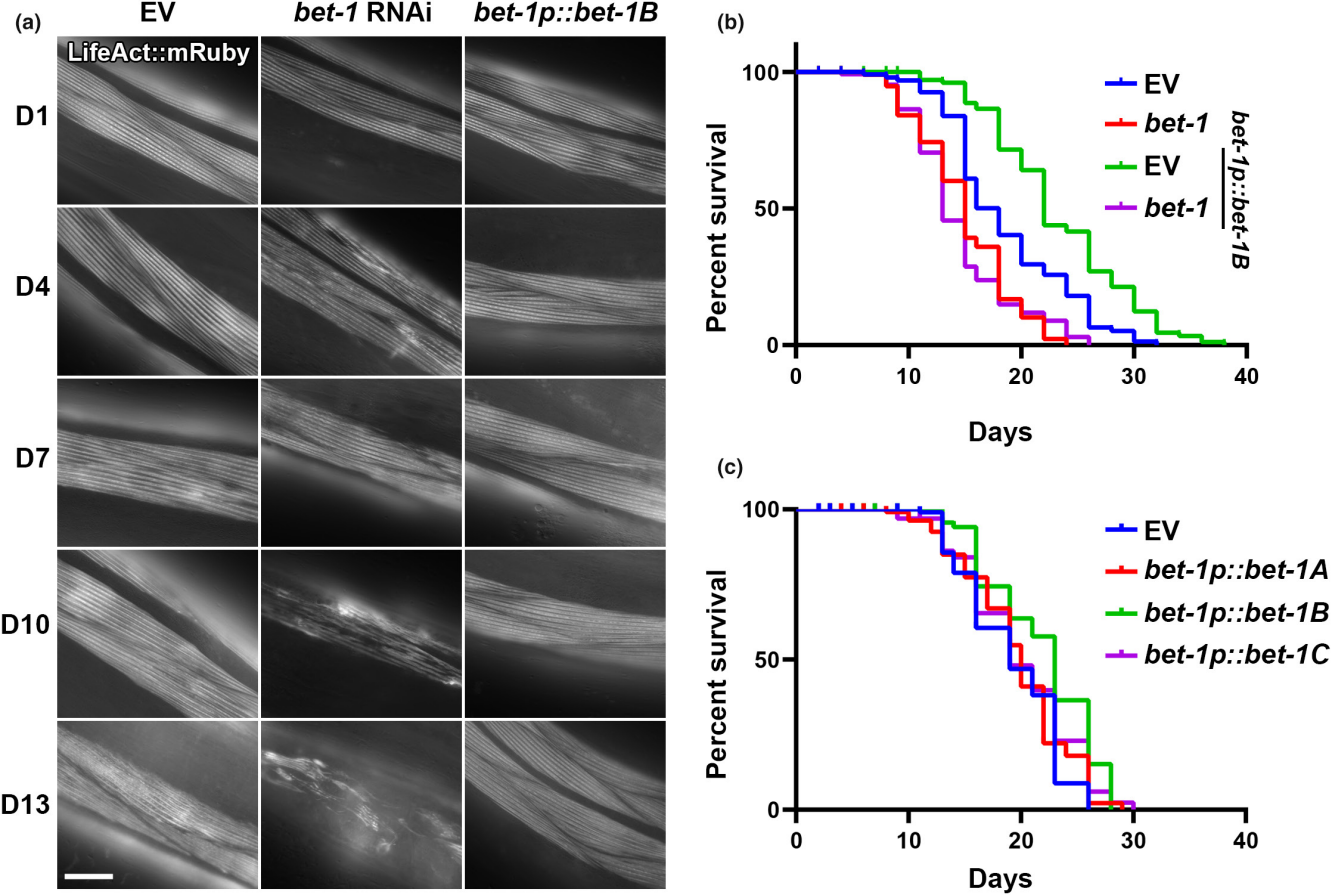
*Bet‐1* expression directly impacts actin function and life span. (a) Representative fluorescent images of adult animals expressing LifeAct::mRuby from a muscle‐specific promoter, *myo‐3p*. N2, wild‐type animals were grown on empty vector (EV) or *bet‐1* RNAi from hatch, and *bet‐1B* overexpression animals (*bet‐1p*::*bet‐1B*) were grown on EV from hatch. All animals were imaged at day 1, 4, 7, 10, and 13 of adulthood. Images were captured on a Zeiss AxioObserver.Z1. Scale bar is 10 μm. (b) Life spans of N2 (blue) and *bet‐1B* overexpression (green) animals grown on EV or *bet‐1* RNAi (N2, red; *bet‐1B* overexpression, purple) from hatch. N2, EV (blue) vs. *bet‐1p*::*bet‐1B*, EV (green) = *p* < 0.0001; N2 *bet‐1* RNAi (red) vs. *bet1p*::*bet‐1*, *bet‐1* RNAi (purple) = not significant. See Tables S6 and S7 for complete life span statistics. (c) Life spans of N2 (EV, blue) and overexpression of *bet‐1A* (*bet‐1p*::*bet‐1A*, red), *bet‐1B* (*bet1p*::*bet‐1B*, array, green), and *bet‐1C* (*bet‐1p*::*bet‐1C*, array, purple) grown on EV from hatch. N2 (blue) vs. *bet‐1p*::*bet‐1B* (green) = *p* < 0.001; N2 (blue) vs. *bet‐1p*::*bet‐1A* (red) or *bet1p*::*bet‐1C* (purple) = not significant. See Tables S6 and S7 for complete life span statistics.

**FIGURE 3:**
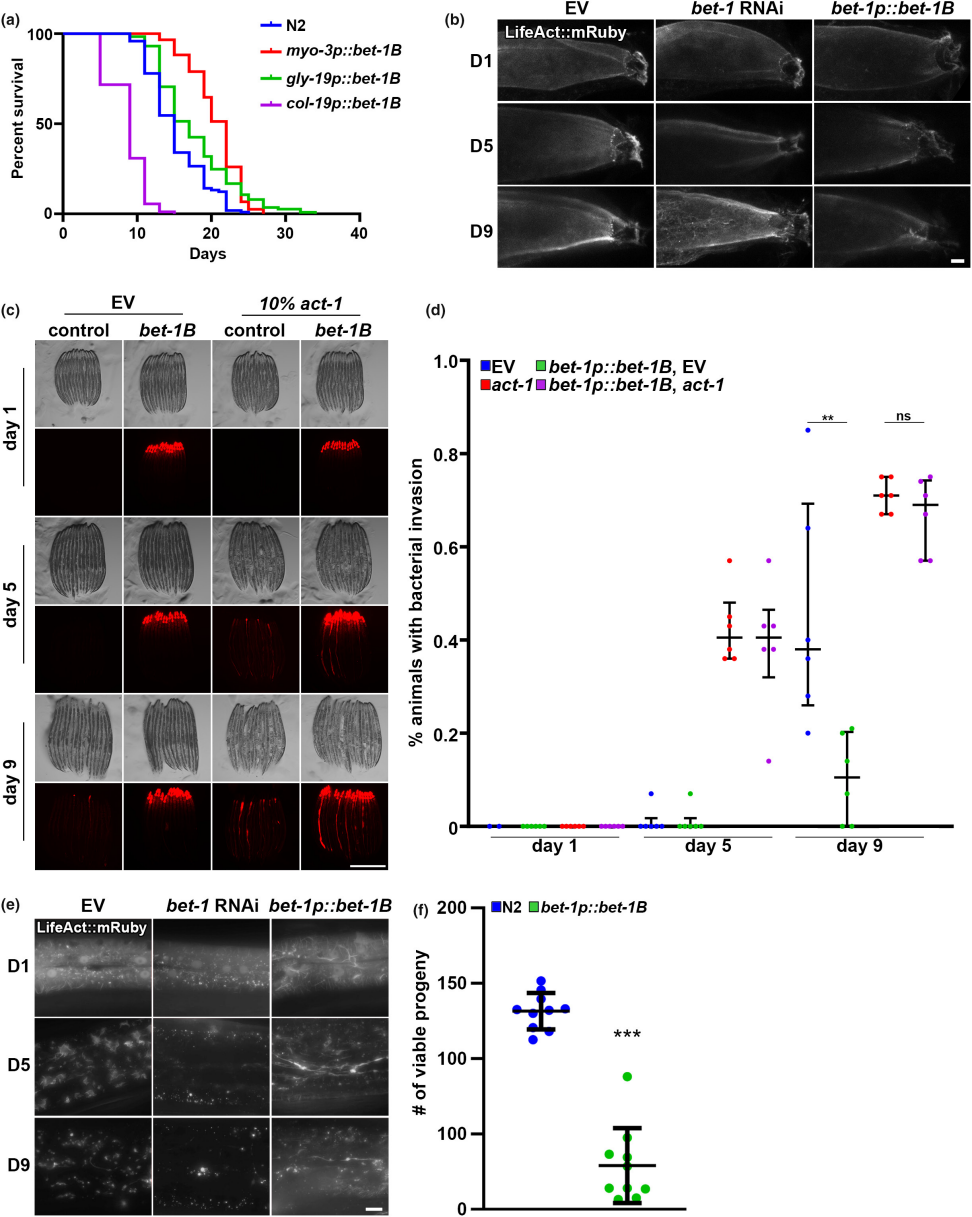
Cell‐type specific effects of *bet‐1B* overexpression. (a) Life spans of N2 (blue) and *bet‐1B* overexpression in muscle (*myo‐3p*, red), intestine (*gly‐19p*, green), and hypodermis (*col‐19p*, purple) grown on empty vector (EV) from hatch. *p*‐Values versus N2 control: *myo‐3p*::*bet‐1B* (red) = *p* < 0.0001; *gly‐19p*::*bet‐1B* (green) = *p* < 0.0001; *col‐19p*::*bet‐1B* (purple) = *p* < 0.0001. See Tables S6 and S7 for complete life span statistics. (b) Representative fluorescent images of adult animals expressing LifeAct::mRuby from an intestine‐specific promoter, *gly‐19p*. N2, wild‐type animals were grown on EV or *bet‐1* RNAi from hatch, and *bet‐1B* overexpression animals (*bet‐1p*::*bet‐1B*) were grown on EV from hatch. All animals were imaged at day 1, 5, and 9 of adulthood. Images were captured on a Leica Stellaris 5 confocal. Scale bar is 10 μm. (c) Representative fluorescent images of adult animals grow on mCherry expressing bacteria as described in Materials and Methods. Wild‐type N2 and *bet‐1B* overexpression (*bet‐1B*) animals were grown on empty vector (EV) or a 90/10 mix of ev/*act‐1* RNAi mixed with 20% mCherry bacteria from hatch. Animals were clarified on OP50 for 2 h prior to imaging at day 1, 5, and 9 of adulthood. Scale bar is 500 μm. (d) Quantification was performed across 2 technical replicates each of 3 biological replicates for a total of 6 replicates. The % of animals exhibiting gut colonization of fluorescent bacteria were quantified per replicate and each dot represents a technical replicate. Lines represent mean and interquartile range. **p < 0.01, n.s. = *p* > 0.05 using student *t*‐test. (e) Representative fluorescent images of adult animals expressing LifeAct::mRuby from a hypodermis‐specific promoter, *col‐19p*. N2, wild‐type animals were grown on EV or *bet‐1* RNAi from hatch, and *bet‐1B* overexpression animals (*bet‐1p*::*bet1B*) were grown on EV from hatch. All animals were imaged at day 1, 5, and 9 of adulthood. Images were captured on a Leica Thunder Imager. Scale bar is 10 μm. (f) Brood assay was measured by counting number of live progeny born by a single parent as described in Materials and Methods in N2 wild‐type (blue) and *bet‐1B* overexpressing (green) animals. Each dot represents a single animal and lines represent median and interquartile range. ***p < 0.001 measured by non‐parametric Mann–Whitney testing. Data is representative of 3 independent trials.

Our data suggest that loss of *bet‐1* results in decreased stability of actin, such that acute exposure to stress is sufficient to perturb actin function. In addition, we find that knockdown of *bet‐1* resulted in the premature breakdown of actin during aging, such that muscle actin showed marked deterioration as early as day 4, compared to wild‐type animals that only start to show mild signs of actin disorganization between day 7–10 (Figure 2a). The RNAi knockdown of *bet‐1*, as validated by reduced transcript levels using RT‐PCR (Figure S3c,d), also caused a decrease in life span (Figure 2b). Surprisingly, overexpression of the primary isoform of *bet‐1*, *bet‐1A*, previously shown to be required for its canonical function in maintenance of cell fate (Shibata et al., 2010) had no impact on life span. Similarly, overexpression of the alternative isoform *bet‐1C* did not impact life span. Instead, overexpression of the *bet‐1* isoform *bet‐1B* significantly increased life span (Figure 2c), which was reversed by *bet‐1* RNAi, suggesting that this is not due to an off‐target effect (Figure 2b). Importantly, overexpression of *bet‐1B* also protected actin filaments at advanced age, as animals with *bet‐1B* overexpression display no measurable changes to actin filaments as late as day 13 when wild‐type animals show obvious collapse (Figure 2a). This increase in actin filament stability also results in increased muscle function, which can be measured by increased motility both at young and old age (Figure 6b, see blue and green), in agreement with a previous study, which described a premature loss of motility in *bet‐1* loss of function animals (Fisher et al., 2013). Finally, we find that overexpression of *bet‐1B* solely in muscle cells is sufficient to result in a significant increase in life span (Figure 3a). These data provide direct evidence that *bet‐1* is a bona fide regulator of actin function in muscle cells, which has direct implications in organismal health and longevity. Importantly, *bet‐1A* overexpression, which did not extend life span, had no effect on actin integrity at late age (Figure S4), adding additional support to the model that protecting actin filaments is correlated with increased longevity.

To determine whether the effects of BET‐1B on actin function were limited to the muscle, we next measured the impact of *bet‐1B* overexpression on actin organization in the intestine and hypodermis, which both show breakdown during aging similar to muscle cells (Higuchi‐Sanabria et al., 2018). RNAi knockdown of *bet‐1* results in premature breakdown of the actin cytoskeleton in intestinal cells (Figure 3b), which is generally visualized as mislocalization of LifeAct away from the microvilli and accumulation in the cytoplasm (Egge et al., 2019). Consistently, we see a decrease in LifeAct accumulation at day 9 in the cytoplasm in *bet‐1B* overexpressing animals compared to wild‐type animals, suggesting that actin integrity is preserved. To determine whether this increase in actin integrity is correlated with increased actin function, we next tested the effect of *bet‐1B* overexpression on gut barrier integrity. The intestinal lining of *C. elegans* is protected by actin‐mediated tight junctions, and the breakdown of this barrier due to agedependent loss of actin function results in bacterial colonization of the gut (Egge et al., 2019). Indeed, we find that *bet‐1B* overexpression protects animals from bacterial colonization at late age (Figure 3c,d). Finally, overexpression of *bet‐1B* solely in the intestine is sufficient to result in a mild, but significant life span extension (Figure 3a).

Surprisingly, in complete opposition to effects in the muscle and intestine, we find that *bet‐1B* overexpression in the hypodermis results in a decrease in life span (Figure 3a). Therefore, we imaged actin filaments in the hypodermis and see *bet‐1* RNAi results in complete loss of actin filaments in the hypodermis. However, *bet‐1B* overexpression results in formation of filaments, which is an unusual structure in the hypodermis, which normally show star‐like structures (Figure 3e). In our model, we posit that overexpression of *bet‐1B* results in hyperstabilization of actin filaments. While this may be beneficial for cells like the muscle or intestine, it is possible that this may be detrimental to the hypodermis where actin is more dynamic (Higuchi‐Sanabria et al., 2018). To further test this hypothesis, we determined the impact of *bet‐1B* overexpression on the only cells that are proliferating in adult animals, the gametes and embryos, as cell division is a process that requires dynamic reorganization of actin. As expected, *bet‐1B* overexpression animals display a significant decrease in brood size (Figure 3f), which suggests that cell division, a process highly dependent on actin dynamics, may also be disrupted in animals with *bet‐1B* overexpression.

**FIGURE 4:**
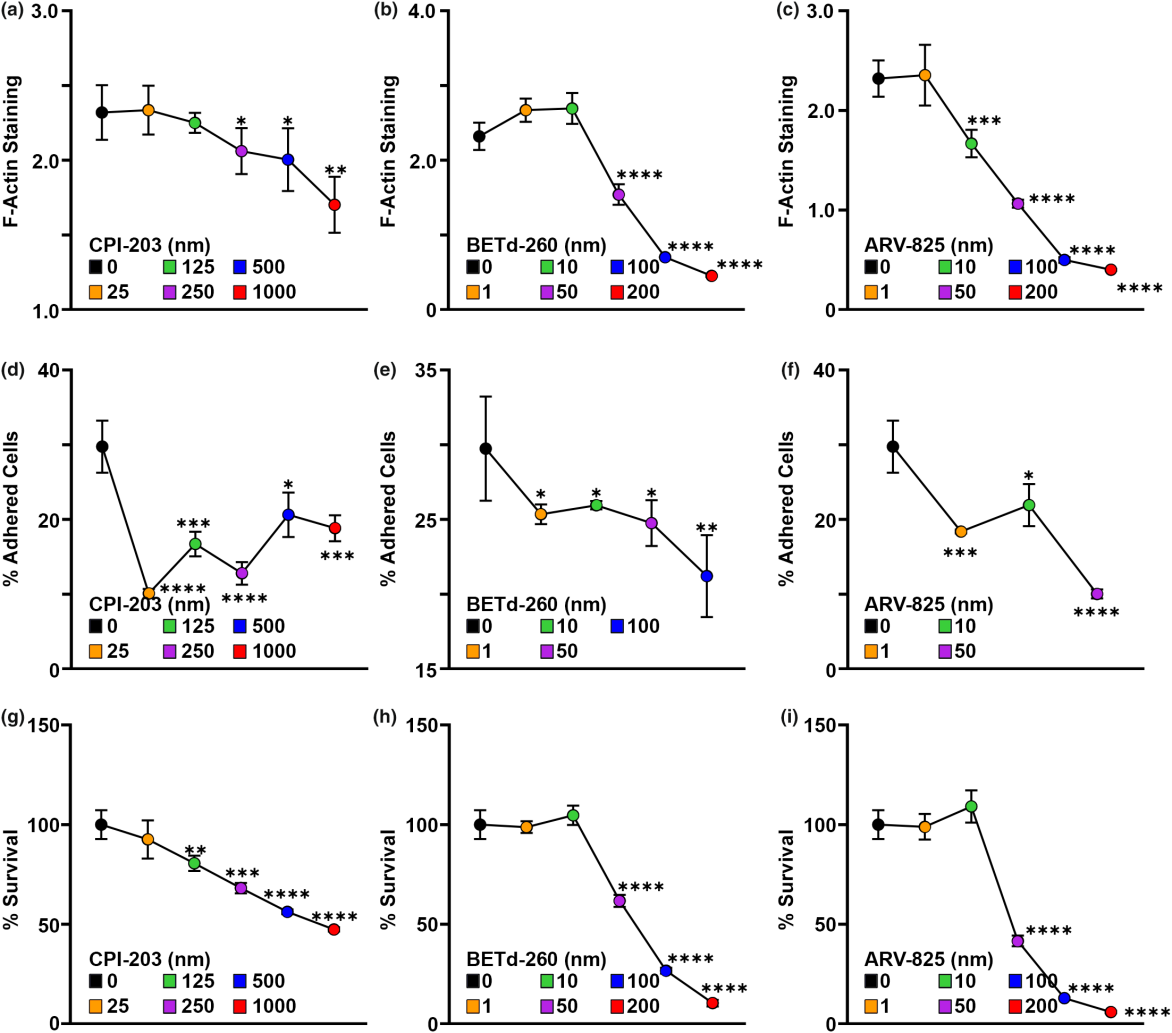
BRD4 Is required for maintenance of F‐Actin and adherence in senescent cells. All measurements were made in IMR‐90 senescent cells produced by etoposide treatment as described in Materials and Methods. F‐Actin filaments were stained with 165 nM Alexa Fluor 488 Phalloidin and integrated fluorescence of F‐Actin staining was normalized against nuclear staining via DAPI in cells treated with varying concentrations of CPI‐203 (a), BETd‐260 (b), and ARV‐825 (c) to inhibit BRD4. Adherence was measured by the fluorescence from the Calcein AM labeled cells and % of adherent cells were measured in cells treated with CPI‐203 (d), BETd‐260 (e), and ARV‐825 (f). % Survival was measured using MTS assay and measured in cells treated with CPI‐203 (g), BETd‐260 (h), and ARV‐825 (i). Dots represent mean and lines are standard deviation across 3 replicates. *p < 0.05; **p < 0.01; ***p < 0.001; ****p < 0.0001 using student *t*‐test.

Next, we sought to determine whether effects of BET‐1 on cytoskeletal function was conserved in human cells. Considering the identification of BRD4/BET‐1 in our initial CRISPR‐Cas9 screen, we were confident that BRD4 is important for sensitivity to the actin destabilizing agent, cytochalasin D. Moreover, taking into account our data on the differences between stable versus dynamic actin in various cell types, studying BRD4/BET‐1 function in dividing cells may have several confounding factors. Therefore, we turned to a senescent cell model. Here, we used human IMR‐90 cells treated with the DNA damaging agent, etoposide (50 μM) for 24 h to induce senescence (Vizioli et al., 2020). Similar to *C. elegans* adult tissue, senescent cells are nondividing cells. Moreover, senescent cells require a stabilized actin network to maintain adherence, which is critical for cell survival (Shin et al., 2020). Therefore, we inhibited BRD4 function in senescent cells using three methods: a chemical BET inhibitor (CPI‐203) (Liang et al., 2019), and the BET proteolysis targeting chimeras, BETd‐260 (Zhang et al., 2019) and ARV‐825 (He et al., 2022), which cause cleavage and degradation of BET proteins. Similar to *C. elegans* muscle and intestine, we see that inhibition of BRD4 in nondividing human cells show destabilization of actin as visualized by decreased F‐actin staining (Figure 4a–c). Moreover, this resulted in a loss of adherence of cells (Figure 4d‐f) and increased senescent cell death (Figure 4g–i). These data suggest that the impact of BET‐1/BRD4 on actin function is conserved in human cells.

### 2.3 BET‐1 affects actin as a transcriptional regulator

BET‐1 is a conserved double bromodomain protein that functions as a transcriptional regulator to maintain stable cell fates (Shibata et al., 2010). Specifically, it recognizes lysine residues of histone tails acetylated by the MYST family of acetyltransferases (MYST HATs), and loss of *mys‐1* and *mys‐2* results in mislocalization of BET‐1 and loss of BET‐1 function (Shibata et al., 2010). To determine whether the functional role of BET‐1 in aging and cytoskeletal maintenance were similar to those of cell fate decisions, we first synthesized a GFP‐tagged variant of BET‐1B. Indeed, GFP::BET‐1B also localize primarily to the nucleus and creates distinct punctae, consistent with previous studies (Figure S5a) (Shibata et al., 2010). In contrast to cell fate decisions, *mys‐1* RNAi was not sufficient to fully phenocopy loss of *bet‐1*, as animals did not exhibit premature deterioration of actin organization (Figure S6a) despite having a comparable decrease in life span, which were not additive with *bet‐1* knockdown (Figure S5b). This may be due to less effective knockdown of *mys‐1* via RNAi (Figure S3c,e). Indeed, previous studies have shown that *mys‐1* is an essential gene and its knockout causes lethality (Shibata et al., 2010), whereas RNAi knockdown of *mys‐1* did not exhibit lethality even when performed from hatch. Alternatively, it is possible that *mys‐1* knockdown has pleiotropic effects as altering histone acetylation status is likely to affect more than just BET‐1 function. However, RNAi knockdown of *mys‐1* suppressed the life span extension and protection of the cytoskeleton at late age found in *bet‐1B* overexpression animals (Figure S6), which does suggest that although some differences exist, similar to cell fate decisions, BET‐1 promotes actin integrity and life span downstream of MYST HATs, likely as a transcriptional regulator.

**FIGURE 5:**
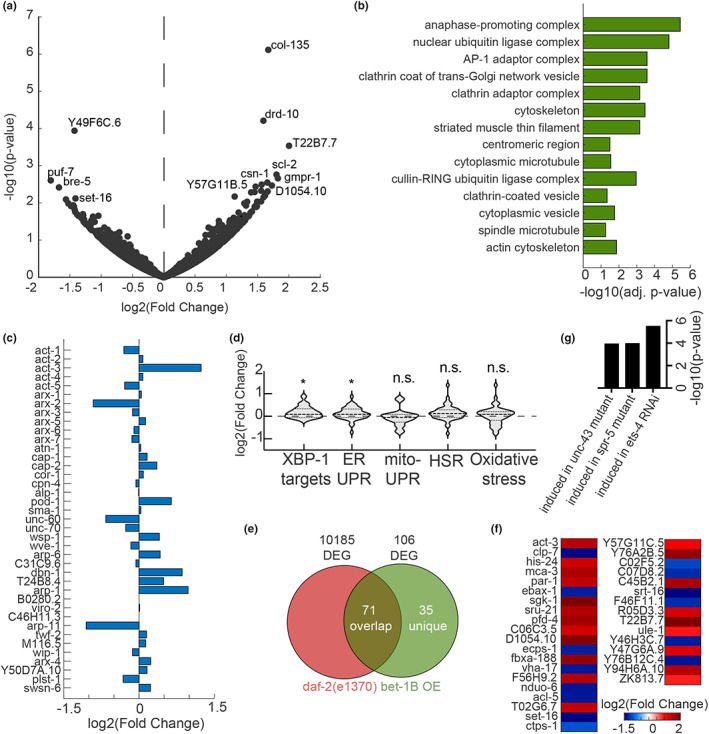
Overexpression of *bet‐1B* drives changes in cytoskeletal regulatory genes. (a) Differentially expressed genes upon overexpression of *bet‐1B*: Volcano plot of the genome‐wide changes in gene expression upon overexpressing *bet‐1B*, as compared to an N2 wild‐type control. (b) Gene ontology enrichments (Chen et al., 2013) for differentially expressed genes (*p*‐value < 0.05) in worms overexpressing *bet‐1B*. (c) log2(fold changes) for all genes annotated as cytoskeleton: Actin function in WormCat (Holdorf et al., 2020). (d) Gene expression changes in groups of genes linked to longevity: endoplasmic reticulum UPR (UPR^ER^) GO:0030968; mitochondrial UPR (UPR^MT^) GO:0034514; heat‐shock response (HSR) GO:0009408; oxidative stress response GO:0006979; and XBP‐1 targets as previously defined (Urano et al., 2002). (e) Comparison of differentially expressed genes (*p*‐value < 0.05) in *bet‐1B* overexpressing worms as compared to a long‐lived *daf‐2(e1370)* mutant (Zarse et al., 2012) reveals a group of 35 unique genes, plotted in (f) see Table S5 for all differentially expressed genes. (g) WormExp (Yang et al., 2016) analysis integrating previously published datasets reveals significant enrichment for the annotated perturbations.

To directly test a functional role for BET‐1 in transcriptional regulation of cytoskeletal gene programs, we performed RNA‐seq in *bet‐1* loss of function and *bet‐1B* overexpression animals. We used both RNAi knockdown and the newly generated *bet‐1* mutant. As expected, we observed a high correlation between the expression profiles of the knockdown and knockout of *bet‐1* (Figure S7a–c). However, since *bet‐1* RNAi targets all isoforms of *bet‐1*, we focused our analysis on the *bet‐1B* overexpression (Figure 5a) and found that genes associated with the general cytoskeleton and the actin cytoskeleton were among the most enriched GO terms (Figure 5b), similar to enrichments observed in the initial human fibroblast screen (Figure S2). Other enriched terms included microtubules, chromatin, and vesicle‐associated terms. Congruent with our observations from our cytoskeletal imaging data, these data suggest that BET‐1‐mediated longevity was through its effects on the cytoskeleton. Therefore, we further focused on all genes related to actin function as previously annotated (Holdorf et al., 2020). We found that out of the five actin genes in *C. elegans*, the expression of *act‐3* was significantly induced. Furthermore, we observed additional changes, including induction and repression, of genes involved in the actin cytoskeleton (Figure 5c), with many opposing effects between the loss of function and *bet‐1B* overexpression datasets across different cytoskeleton‐related gene groups (Figure S7d). Strikingly, these transcriptional changes were dependent on *mys‐1*, as *mys1* knockdown reversed most of the induction and repression of differentially expressed genes, including the induction in *act‐3* (Figure S7e,f). These data are consistent with a model whereby BET‐1 impacts life span and actin function through its role as a transcriptional regulator downstream of MYS‐1.

We then tested whether other protective pathways that have been linked to longevity were altered upon *bet‐1B* activation. We could not find activation of the mitochondrial unfolded protein response (UPR^MT^), the heat‐shock response (HSR), or oxidative stress response. We did observe a mild activation of genes involved in the endoplasmic reticulum UPR (UPR^ER^). These data were further verified by direct comparison with a dataset identifying targets of the UPR^ER^ transcription factor, XBP‐1 (Figure 5d). Consistent with these findings, we find that the life span extension we find in *bet‐1B* overexpressing animals is dependent on the major UPR^ER^ regulator, XBP‐1, as RNAi knockdown of *xbp‐1* fully suppressed the life span extension of these animals (Figure S8a). We next tested whether *bet‐1B* overexpression directly impacts ER stress resilience, as UPR^ER^ induction has previously been shown to increase resistance to tunicamycin, which induces ER stress by blocking N‐linked glycosylation (Daniele et al., 2020; Higuchi‐Sanabria et al., 2020; Taylor & Dillin, 2013). Similar to *xbp‐1s* overexpression, *bet‐1B* overexpression significantly increased ER stress resilience (Figure S8b), suggesting that a functional UPR^ER^ is also activated by BET‐1B.

To further test the response of *bet‐1B* overexpressing animals to stress, we next measured the sensitivity of these animals to paraquat, which induces synthesis of reactive oxygen species in the mitochondria to induce mitochondrial and oxidative stress (Castello et al., 2007). Interestingly, we find that *bet‐1B* overexpression results in a significant increase in resistance to paraquat treatment (Figure S8c). However, since paraquat induces both oxidative and mitochondrial stress, we next measured the effect of *bet‐1B* overexpression on response to mitochondrial stress using the UPR^MT^ reporter, *hsp‐6p*::*GFP* (Yoneda et al., 2004) and to oxidative stress using the SKN‐1 reporter, *gst‐4p*::*GFP* (Link & Johnson, 2002). Interestingly, we find that *bet‐1B* overexpression has no impact on either basal or stress‐induced oxidative stress response (Figure S8d). However, we did see that *bet‐1B* overexpression resulted in a mild increase in stress‐induced UPR^MT^ (Figure S8e), suggesting that *bet‐1B* overexpressing animals are better able to induce UPR^MT^ under stress, which may be the cause of the increased resistance to paraquat.

We also compared our dataset to a previously published gene expression dataset of *daf2(e1370)* (Zarse et al., 2012), a mutant with a reduced function of the insulin/IGF‐1 receptor signaling, which has been implicated in aging. We found that 71 out of 106 differentially expressed genes overlapped between worms overexpressing *bet‐1B* and the long‐lived *daf2(e1370)* (Figure 5e). Consistent with these findings, the life span extension of *bet‐1B* overexpressing animals is also fully dependent on *daf‐16* (Figure S9a), which encodes the key longevity transcription factor in response to insulin/insulin‐like growth factor signaling and is critical for the extreme life span extension of *daf‐2* mutant animals (Murphy et al., 2003). Finally, we tested whether the life span promoting effects of *bet‐1B* overexpression were dependent on HSF‐1, the heat‐shock transcription factor, as overexpression of HSF‐1 has previously been shown to recruit DAF‐16 (Douglas et al., 2015). Moreover, *hsf‐1* overexpression has also been shown to promote actin function during aging (Baird et al., 2014; Higuchi‐Sanabria et al., 2018). Shockingly, we find that *bet‐1B* overexpressing animals still significantly extend life span, even in the absence of *hsf‐1* (Figure S9b), suggesting that the mechanisms of action of BET‐1 and HSF‐1 are distinct.

### 2.4 The beneficial effects of *bet‐1B* overexpression are dependent on effects on Actin

Among the genes unique to the *bet‐1B* overexpressing worms was *act‐3* (Figure 5f). Interestingly, our investigation of genes that were significantly induced in the *bet‐1B* overexpressing worms showed a similar transcriptional signature to that induced by knockdown of the transcription factor *ets‐4/SPDEF* (erythroblast transformation specific/SAM pointed domain containing ETS transcription factor), which has been previously linked to aging (Thyagarajan et al., 2010), potentially through regulation of the actin cytoskeleton via VASP (vasodilator‐stimulated phosphoprotein) (Bear et al., 2002; Ye et al., 2020) (Figure 5g). In addition, we also observed a similarity with genes affected by knockdown of the methyl demethylase *spr‐5* (suppressor of presenilin), another gene whose reduction has been linked to longevity (Greer et al., 2016). Together, these data provide further evidence that the pro‐longevity transcriptional program induced by *bet‐1B* is at least in part due to a dedicated program to promote cytoskeletal health.

**FIGURE 6:**
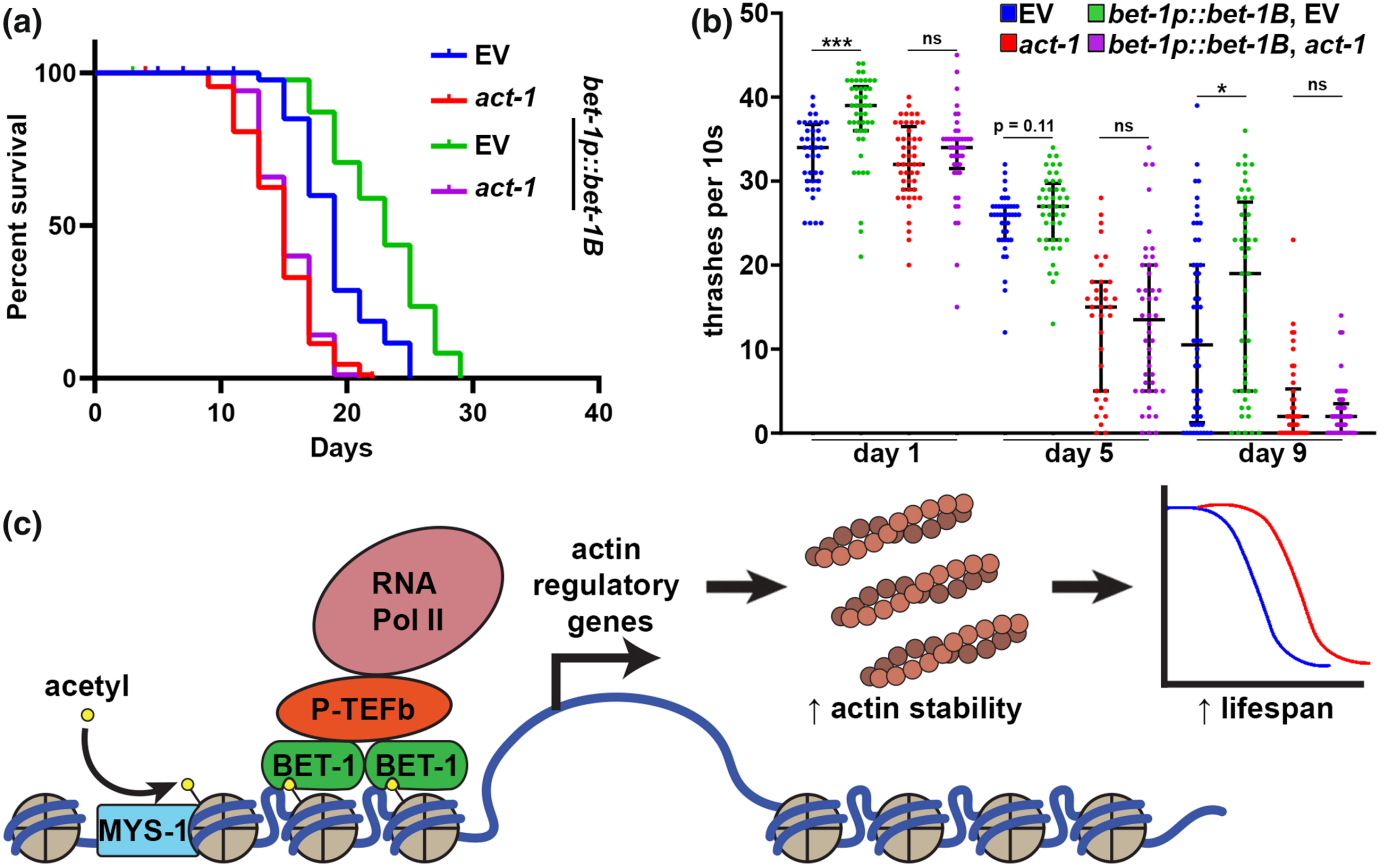
*Bet‐1* overexpression promotes life span and healthspan through effects on actin. (a) Life spans of wild‐type, N2 (EV, blue) and *bet‐1b* overexpression (*BET‐1p*::*bet‐1B*, green) animals grown on EV or *10% act‐1* RNAi (N2, red; *bet‐1B* overexpression, purple) from hatch. N2, EV (blue) vs. *bet‐1p*::*bet‐1B*, EV (green) = *p* < 0.0001; N2 *act‐1* RNAi (red) vs. *bet‐1p*::*bet‐1B*, *act‐1* RNAi (purple) = not significant. See Tables S6 and S7 for life span complete statistics. (b) Thrashing assays were performed on N2 (EV, blue) and *bet‐1B* overexpression (*bet‐1p*::*bet‐1B*, green) animals grown on EV or *10% act‐1* RNAi (N2, red; *bet‐1B* overexpression, purple) from hatch. Animals were grown on FUDR to prevent progeny development and assayed on day 1, 5, and 9 of adulthood. Recordings were performed in M9 solution on a Leica M205FCA stereomicroscope with a Leica K5 camera and thrashing was scored manually over a 10 s recording. Data is representative of three independent trials. *n* = 36–54 worms per condition. ***p < 0.001; *p < 0.05; ns = *p* > 0.05 calculated using non‐parametric Mann–Whitney testing. Each dot represents a single animal and lines represent median and interquartile range. (c) Model for BET‐1 regulation of Actin. MYS‐1 acetylates histones (Ceol & Horvitz, 2004), allowing recruitment of BET‐1 to chromatin (Shibata et al., 2010). BET‐1 recruitment to chromatin allows for recruitment of transcription initiation factors like P‐TFEb, which allows recruitment of RNA pol II (Jang et al., 2005) to promote transcription of Actin regulatory genes, which promotes Actin function and life span.

To experimentally validate our findings from transcriptome analysis, we tested the impact of destabilizing the actin cytoskeleton on the beneficial effects of *bet‐1B* overexpression. Importantly, perturbations of actin function completely suppressed the life span extension found in *bet‐1B* overexpression animals (Figure 6a). Specifically, actin function was perturbed by 10% *act‐1* RNAi, similar to conditions used for our synthetic lethality screens. 10% *act‐1* RNAi results in a significant decrease in life span extension, and overexpression of *bet‐1B* has no impact on life span in these animals. Thus, the life span extension of *bet‐1B* overexpression is indeed dependent on its alterations of the actin cytoskeleton, as knockdown of actin was sufficient to reverse the longevity phenotype. Similarly, perturbation of actin function suppressed the beneficial effects of *bet‐1B* overexpression on motility (Figure 6b) and gut barrier function (Figure 3c,d). These data suggest that the beneficial effects of *bet‐1B* are primarily due to its role in promoting cytoskeletal function, likely as a transcriptional regulator.

## 3 DISCUSSION

The actin cytoskeleton is a complex and dynamic cellular organelle, which requires a tight regulation of the biosynthesis of its building blocks, their polymerization, disassembly, and breakdown. The dynamic rearrangements of actin underlie many cellular functions, and have critical implications on organismal phenomena, such as disease states and overall health. Many previous works focused on understanding the interactions between the players which directly take part in the cytoskeleton. For example, a competition model has been proposed to exist between actin assembly factors and monomeric actin (Davidson & Wood, 2016). Inspired by the protein homeostasis pathways of other organelles (Dutta et al., 2022), we were intrigued to explore whether a master transcriptional regulator may exist, which drives and promotes cytoskeletal health. Interestingly, our cross‐species screen revealed potentially evolutionarily conserved regulators of actin homeostasis between mammals and *C. elegans*. Through CRISPR‐Cas9 screening in karyotypically normal human fibroblasts, we identified genes that when knocked out impact survival under actin destabilization caused by exposure to cytochalasin. Cytochalasin D is a mycotoxin that inhibits actin polymerization by binding to F‐actin and preventing polymerization of actin monomers (May et al., 1998). Gene ontology analysis of our significantly depleted genes identified actin cytoskeletal organization as the highest enriched gene set, as well as genes involved in cell‐matrix adhesion, cell motility, wound healing, intracellular transport, and autophagy. These pathways all mechanistically require a properly functional actin cytoskeleton, which gave us high confidence in our initial screen. However, a few limitations of our screen are that we used the Avana sgRNA KO library, which since its production has been outperformed by some other libraries, especially CRISPR interference (CRISPRi) or CRISPR activation (CRISPRa) platforms, which can enable more flexible gene modulation (Sanson et al., 2018). In addition, our utilization of cytochalasin D can be limiting due to the wide‐range effects of the drug (Foissner & Wasteneys, 2007).

Rather than optimize our primary screen with alternative libraries or other actin‐destabilizing drugs (e.g., latrunculin), we opted for a secondary screening platform in *C. elegans*. We reasoned that as one of the most highly conserved genes/proteins across eukaryotes in sequence and function, a cross‐species approach would be optimal to identify critical regulators of actin. In addition, *C. elegans* are an in vivo whole animal model, free from some of the restrictions or caveats of an in vitro cell culture system, such as major changes to actin cytoskeletal integrity when grown in a plastic dish (Tharp et al., 2021). Finally, phenotypic analysis in *C. elegans* is not limited solely to growth rate, and has multiple levels of phenotyping, including development, motility, fecundity, size, and overall physiological health, all of which can be rapidly screened simultaneously. For our secondary screen, we used a genetic screening method whereby we performed partial knockdown of all actin genes using RNAi. We opted for this method as the thick cuticle of *C. elegans* lead to low permeability of many drugs (Xiong et al., 2017), making the usage of cytochalasin D not only cost‐prohibitive for this study, but low confidence in maintaining a homogenous effect. Moreover, the actin cytoskeleton is important for cuticle development in worms (Costa et al., 1997) further compounding on this problem, and thus screening for regulators of actin using a drug may introduce several caveats that can bias hits for those involved in cuticle development. Overall, our two‐species screening platform attempted to make use of two robust platforms equipped with a unique set of benefits that allowed for identification of evolutionary‐conserved regulators of actin.

Through our cross‐species approach, we identified *bet‐1*, the *C. elegans* ortholog of human BRD2 and BRD4. *bet‐1* encodes a double bromodomain protein that has been originally characterized for its role in cell fate decisions (Shibata et al., 2010). In addition, BET‐1 has been found to directly impact muscle myosin levels during aging, likely as a transcriptional regulator (Fisher et al., 2013). Actin filaments are usually in direct association with myosin (Cooper, 2000) and actin‐myosin interactions are critical for *C. elegans* body wall muscle structure and function (Gieseler et al., 2017). Moreover, BRD4 has been implicated in cancer aggression as an angiogenesis promoting factor, a process that directly involves actin polymerization (Huang et al., 2016). Interestingly, BRD2 (an alternative homolog of *bet‐1*) dosage has been previously linked to longevity in C57B6/J mice, although the underlying molecular basis was not identified (Pathak et al., 2020). While these previous studies showed potential correlation of BET1/BRD2/BRD4 with actin function and/or longevity, our study has now provided evidence that expression of *bet‐1* directly impacts actin organization and function, which has direct significance in longevity. Specifically, loss of function of *bet‐1* results in premature breakdown of actin structure during aging, while its overexpression protects actin filaments at late age and promotes both healthspan and life span. Importantly, we find that these effects are conserved in human cells, as inhibition of BRD4 in non‐dividing, human senescent cells result in decreased actin filaments, decreased adhesion, and decreased cell survival.

What was intriguing was that BET‐1's impact on actin function is seemingly context dependent. We found that *bet‐1B* overexpression protected actin filaments in the muscle and intestine, which promoted longevity, but had negative effects on the hypodermis. Our prediction in this discrepancy across cell types is that the primary requirement of actin is different in each cell. For example, in the muscle, actin is required for contractile movement of the muscle along myosin filaments, and muscle actin structures are highly stable (Higuchi‐Sanabria et al., 2018). Similarly, intestinal actin structures are highly stable and make up the gut barrier (Egge et al., 2019). However, actin in the hypodermis is required for very dynamic processes, including wound healing (Xu & Chisholm, 2011) and endocytosis (Higuchi‐Sanabria et al., 2018). Thus, it is entirely possible that overexpression of *bet‐1B* promotes actin stability, which might be detrimental to cells like the hypodermis where it is imperative for actin to remain highly dynamic. Consistent with this hypothesis, we also found that *bet‐1B* overexpression significantly decreased reproduction, a process highly dependent on actin turnover for cell division. Our data suggests that *bet‐1B* promotes actin stability, which will benefit highly stable actin environments (e.g., muscle and intestinal actin), but may be detrimental where actin dynamics are required (e.g., dividing cells and hypodermis).

Since we see that all visible BET‐1B protein is found within the nucleus and localizes to puncta which in no way resembles actin filaments, we believe that BET‐1B does not impact actin through direct interactions. Moreover, the beneficial effects of BET‐1B on actin and life span are dependent on MYS HATs, which acetylate histones at specific lysine residues, allowing BET‐1B binding to alter gene expression (Shibata et al., 2010, p. 1). Thus, BET‐1B likely impacts actin function as a transcriptional regulator that promotes expression of actin regulatory genes (Figure 4c). Indeed, our transcriptome analysis revealed that cytoskeletal regulators make up a large majority of upregulated genes in *bet‐1* overexpressing animals, with *act‐3* specifically being highly induced. We also observed changes in other cytoskeletal genes, including different regulatory factors. These genes showed opposing effects when compared to *bet‐1* loss of function animals and were largely dependent on the HAT, *mys‐1*. Importantly, the transcriptional changes induced by BET‐1B were largely distinct from the UPR^MT^, HSR, and the oxidative stress response.

However, we did observe a mild overlap with UPR^ER^ genes and a mutant with reduced *daf‐2* function. In addition, we found that the beneficial effects of *bet‐1B* overexpression on life span were dependent on the UPR^ER^ component, XBP‐1. These findings were not entirely surprising as XBP‐1 has previously been shown to regulate cytoskeletal targets (Shen et al., 2005), which suggests that perhaps there are some overlaps between UPR^ER^ and actin regulation. Consistent with these findings, *bet‐1B* overexpression also significantly increased resilience to ER stress. Of future interest would be to determine whether *bet‐1B* improves ER stress resilience solely by activating UPR^ER^ components, or whether actin health and function has direct ramifications in ER quality control and/or ER stress resilience. In other instances, actin function has been correlated with ER dynamics: for example, increases in intracellular calcium levels, which induces polymerization of actin at the ER (Wales et al., 2016) can have direct effects on ERmitochondrial contacts to drive mitochondrial dynamics (Chakrabarti et al., 2018; Korobova et al., 2013). Interestingly, we also found that *bet‐1B* overexpression improved resistance against paraquat, which induces both mitochondrial and oxidative stress by promoting synthesis of superoxide in the mitochondria. However, we found that this was likely due to increases in UPR^MT^ induction rather than improved oxidative stress resilience. Overall, these data beg the question of whether improved actin homeostasis through BET‐1B can improve overall organismal stress resilience and a tantalizing hypothesis that BET‐1B drives an Actin Cytoskeletal Stress Response (ACSR), which can impact organismal health. An exciting area of future work is to investigate whether a BET‐1B driven ACSR – possibly in coordination of other cytoskeletal regulators including DAF‐16 and HSF‐1 – can drive overall stress resilience and longevity.

Finally, we found that the life span extension of *bet‐1B* overexpression was dependent on the insulin/IGF‐1 transcription factor, DAF‐16. While DAF‐16 itself has not been directly tied to cytoskeletal function, FOXO transcription factors have direct impacts on actin function in mammalian systems (Birnbaum et al., 2019), and this is likely a conserved phenomenon in *C. elegans*. Despite these overlaps, a third of the genes induced by BET‐1 were unique and included cytoskeletal and chromatin‐related genes, suggesting that at least part of BET‐1's transcriptional program is independent of DAF‐2/DAF‐16.

In both mammals and *C. elegans*, BET‐1/BRD4 has been shown to directly bind chromatin (Dey et al., 2000; Floyd et al., 2013; Shibata et al., 2010) and play an active role in gene expression (Fisher et al., 2013; Lovén et al., 2013). In mammals, BRD4 has direct chromatin decompaction activity. Specifically, BRD4 can act as a HAT to directly acetylate histones H3 and H4, resulting in nucleosome clearance and chromatin decompaction (Devaiah et al., 2016). BRD4 can also recruit transcription super‐complexes that promote RNA‐PolII activity to stimulate transcription (Donati et al., 2018). While mammalian research has progressed in characterizing a mechanism whereby BRD4 impacts transcription, work on how BET‐1 modulates transcription in *C. elegans* has yet to be discovered. Since both BRD4 and BET‐1 bind chromatin at histones acetylated at similar lysine residues (H3 K14 or H4 K5/K12 in mammals (Chiang, 2009; Dey et al., 2000); H4 K5, K8, K12, and K16 in *C. elegans* (Shibata et al., 2010)) through conserved bromodomains, it is feasible that the mechanisms of action are similar between mammals and worms. However, additional work is necessary to uncover the direct mechanism whereby BET‐1 modulates transcription.

Together, our cross‐species approach identified a unique function for BET‐1 in actin cytoskeletal maintenance during aging, likely through a conserved function as a transcriptional regulator. Whether BET‐1 can directly sense perturbations to cytoskeleton health, or whether the information is relayed by an upstream cytoplasmic sensor, remains to be explored. These findings assign BET‐1 a key role as a regulator of cytoskeleton homeostasis, possibly linking previous associations with disease states to this underlying cellular function.

## 4 METHODS

### 4.1 Culturing BJ fibroblasts and cytochalasin screen

The immortalized human foreskin fibroblast line BJ ATCC CRL‐2522 (BJ fibroblasts) expressing hTERT and Cas9 were used for the CRISPR‐Cas9 based screen as previously described (Schinzel et al., 2019). Cells were cultured in gelatin‐coated dishes in Dulbecco's modified. Eagle's medium (DMEM), 15% fetal bovine serum (FBS), 1% glutamax, 1% non‐essential amino acids (NEAA), and 1% penicillin/streptomycin. For splitting, cells were washed with PBS, trypsinized, and replated at 1:3 or 1:6 ratios based on confluence. Fresh media was applied every other day.

For the CRISPR‐Cas9 screen, cells were transduced with the AVANA genome‐wide sgRNA lentiviral library (Doench et al., 2016; Shalem et al., 2014) and selected for 2 weeks with puromycin to maximize genome editing and target protein depletion. Cells were then split into a control (DMSO) arm and 0.1 μM cytochalasin D treatment arm and harvested after 2 weeks of treatment for sequencing. Genomic DNA (gDNA) extraction was performed on a frozen cell pellet in a 15 ml conical tube on 3 × 10^7^–5 × 10^7^ cells. Cells were lysed in 50 mM Tris, 50 mM EDTA, 1% SDS, 0.1 mg/ml Proteinase K overnight at 55°C. Then RNAse A was added to a final concentration of 50 μg/ml and incubated at 37°C for 30 min. Next, ammonium acetate was added to a final concentration of 2.5 M to precipitate proteins, samples were vortexed at max speed for 20 s and centrifuged at 4,000 *g* for 10 min. The supernatant was carefully removed and genomic DNA was extracted with cold 100% isopropanol, washed with cold 70% ethanol, air dried for 30 min to remove excess ethanol, and resuspended in Tris‐EDTA. Sequencing was performed using Illumina Next Generation Sequencing as previously described (Shalem et al., 2014). Raw sgRNA counts are provided in Table S5.

CellTiter‐Glo was used to estimate cell density for titration of cytochalasin D as previously described (Schinzel et al., 2019). Briefly, cells were treated with indicated concentrations of cytochalasin D or a DMSO vehicle control. Plates were washed 1x with PBS to eliminate excess media and CellTiter‐Glo media was added to each cell in a 1:3 dilution into cell/PBS mix. Mix was incubated for 30 min at 37°C and luminescence was measured using a Tecan M1000.

For Annexin V staining, BJ fibroblasts were treated with cytochalasin D at 0–10 μM concentrations for 24 h and 3‐h treatment with 30 mM H_2_O_2_ was used as a positive control for cell death. Annexin V FITC (Biolegend, 640906) and live/dead Aqua (ThermoFisher, L34966) staining was performed as per manufacturer's protocol. Briefly, cells were stained with both dyes for 20 min at room temperature in Annexin V binding buffer (Cayman Chemical, 600302). Dyes were washed once with Annexin V binding buffer, resuspended with Annexin V binding buffer, and flow cytometry (Attune, ThermoFisher) was used to quantify staining. For statistical analysis, two‐way ANOVA with Dunnett's test was used to compare the ratio of live cells between the 0 μM control against each drug‐treated group. 3 technical replicates of 2 biological replicates were performed.

### 4.2 Culturing and experiments with senescent cells

Human primary IMR‐90 fibroblasts were grown in DMEM, supplemented with 10% FBS, 100 U/ml penicillin, 100 μg/ml streptomycin, and 2 mM glutamine at 37°C in a humidified atmosphere with 5% CO2 and 3.5% oxygen (Vizioli et al., 2020). For inducing senescence, cells were treated with DNA damaging agent etoposide (50 μM) for 24 h. The etoposide‐media was then replaced with DMSO and BET inhibitor/degrader drug‐containing media. Old media were replaced with new media containing DMSO and drugs every 2–3 days, and cells were harvested after 8 days for cell survival, adhesion, and actin polymerization assay.

To check the cell viability, we performed an MTS assay as per manufacturer's protocol with modifications. Briefly, approximately 12000 IMR90 cells were seeded per well of 96‐well plate. Senescence induction and drug treatments were performed as aforementioned. MTS assay solution was prepared by adding 20 μl CellTiter 96® AQueous One Solution Reagent (Promega, Cat. No. G3580) per 100 μl culture medium. The old medium from senescent IMR‐90 cells was aspirated and 120 μl MTS assay solution per well was added. After 2 h incubation, 100 μl MTS assay solution aspirated from the plate in a fresh 96‐well clear plate and the absorbance at 495 nm was taken immediately using Microplate Reader.

To examine the adhesion capacity of senescent cells after drug treatments, VybrantTM Cell Adhesion Assay Kit (Molecular Probes, Cat. No. V‐13181) was used according to the manufacturer's protocol with modification. Briefly, 30–40% confluent IMR‐90 cells were treated with etoposide and BET inhibitor/degraders drugs in 6‐wells plates as previously mentioned. After 8 days, cells from each well were trypsinized and pelleted down after neutralizing the trypsin with complete media. The cells were washed once with PBS and resuspended in 1 ml serum free DMEM containing 5 μM Calcein AM solution for 30 min. Then cells were washed twice with complete media to remove the excess Calcein AM. Finally, the Calcein AM labeled cells were suspended in 700 μl complete media and 100 μl of suspension was seeded per well in a PhenoPlate‐96 (PerkinElmer). Cells from each treatment were seeded in total 6 wells of 96 wells. After 4 h incubation, 3 wells from each treatment were washed 3 times with PBS to assay the adhering cells and 3 wells were maintained without washing to assay the total cells. Fluorescence was measured using Clariostar Multi‐Mode Microplate Reader using FITC channel. The percentage of adhered cells were calculated from the fluorescence of adhered cells against total cells seeded after background subtraction.

To detect the level of actin polymerization, we used Alexa Fluor 488 Phalloidin (Life Technologies, A12379), because this bicyclic peptide specifically binds to polymerized F‐actin. Approximately 12,000 IMR90 cells were seeded in a PhenoPlate‐96 (PerkinElmer) and treated with etoposide and drugs, as previously mentioned. After 8 days of etoposide treatment, cells were fixed with 4% paraformaldehyde. After washing the fixed cells with PBS three times, cells were permeabilized with 0.2% Triton‐X and blocked with 1% BSA solution in PBS for 30 min to reduce the background staining. After blocking, F‐Actin of the cells was stained with 165 nM Alexa Fluor 488 Phalloidin for 1 h at room temperature. Cells were washed with PBS three times and nuclei were stained with DAPI. The fluorescence of the wells was measured by using Clariostar Multi‐Mode Microplate Reader.

### 4.3 *Caenorhabditis elegans* strains and maintenance

All strains used in this study are derivatives of the N2 wild‐type worm from the Caenorhabditis Genetics Center (CGC) and are listed below. Worms are maintained at 15°C on OP50 *E. coli* B strain and switched to growth at 20°C on HT115 *E. coli* K strain for all experimentation. HT115 bacteria carrying a pL4440 empty vector control or expressing double‐stranded RNA containing the sequence against a specific target gene were used for all experimentation. Experiments are performed on age‐matched animals synchronized using a standard bleaching protocol (Bar‐Ziv et al., 2020). Briefly, animals are collected using M9 solution (22 mM KH_2_PO_4_ monobasic, 42.3 mM Na_2_HPO_4_, 85.6 mM NaCl, 1 mM MgSO_4_) and bleached in 1.8% sodium hypochlorite and 0.375 M KOH diluted in M9 until all carcasses were digested. Intact eggs were then washed 4× with M9 solution followed by L1 synchronization by floating eggs in M9 overnight in a 20°C incubator on a rotator for a maximum of 16 h. Synchronized animals are always grown on standard RNAi plates (1 mM CaCl_2_, 5 μg/ml cholesterol, 25 mM KPO_4_, 1 mM MgSO_4_, 2% agar w/v, 0.25% Bacto‐Peptone w/v, 51.3 mM NaCl, 1 μM IPTG, and 100 μg/ml carbenicillin; HT115 *E. coli* K strain containing pL4440 vector control or pL4440 with RNAi of interest).

For *bet‐1* overexpression, isoforms A, B, and C were defined as per (Shibata et al., 2010) and sequences are provided below. Coding sequences were cloned from cDNA synthesized via reverse transcriptase using RNA isolated from N2 worms, the endogenous *bet‐1* promoter was cloned from gDNA isolated from N2 worms, and an unc‐54 3′UTR was cloned from gDNA isolated from N2 worms. Plasmids were injected into N2 worms using a standard microinjection protocol as described (Garcia et al., 2022) with 10 ng/μl of overexpression plasmid, 2.5 ng/μl of pEK2 (*myo‐2p*::*tdtomato*) as a co‐injection marker, and 100 ng/μl of pD64 vehicle as filler DNA. Worms positive for the fluorescent pharynx were selected to identify stable arrays. Integration was performed by gamma irradiation where L4 worms were irradiated with 4000–4400 rems of radiation and integration events were selected by finding animals that maintained 100% frequency of co‐injection marker in the F3 generation. Lines were then backcrossed into N2 a minimum of 8x to eliminate mutations. For overexpression of *3xHA*::*GFP*::*bet‐1B*, a GFP sequence containing introns and a 3xHA cassette was cloned upstream of the *bet‐1B* coding sequence. Injection and integrations were performed by SUNY Biotech.

To synthesize the *bet‐1(uth41)* mutant line, we used a Cas9‐RNA protocol as published on the IDT website via the Dernberg lab. Briefly, a mixture of 15 μM trRNA, and 22 μM crRNA (accatgggcaagtccgcgac) were incubated for 5 min at 95°C, cooled, then mixed with 24 μM Cas9 protein and left at room temperature for 5°C. 2.5 ng/μl of pEK2 (*myo‐2p*::*tdtomato*) was added as a co‐injection marker and this mixture was injected into *C. elegans* gonads. All progeny positive for the co‐injection marker were selected, then sequenced for INDELs that incorporated a premature stop codon. The *bet‐1(uth41)* mutant has a premature stop codon at amino acid 17.

**Table:** 

Strains used in this study		
*C. elegans*: Bristol (N2) strain as wild‐type (WT)	CGC	N2
*C. elegans*: CL2166: dvIs19[pAG15(gst4p::GFP::NLS)] III	CGC	
*C. elegans*: AGD1988: zcIs13[hsp‐6p::GFP]	This study	SJ4100 6x backcross
*C. elegans*: AGD2051: bet‐1(uth41) I	This study	N/A
*C. elegans*: AGD2161: uthIs505(bet‐1p::bet1A::unc‐54 UTR; myo‐2p::tdTomato)	This study	N/A
*C. elegans*: AGD2414: uthIs 498 (bet‐1p::bet1B::unc‐54 UTR; myo‐2p::tdTomato)	This study	N/A
*C. elegans*: AGD2498: unc‐119(ed3) III; uthSi7[myo‐3p::LifeAct::mRuby::unc‐54 3′UTR::cbunc‐119(+)] IV; uthIs498 (bet‐1p::bet‐1BCDS::unc54 UTR; myo‐2p::tdTomato)	This study	N/A
*C. elegans*: AGD2332: uthEx907(bet‐1p::bet1C::unc‐54 UTR; myo‐2p::tdTomato)	This study	N/A
*C. elegans*: AGD2936: uthIs498[bet‐1p::bet1b(CDS)::unc‐54 UTR; myo‐2p::tdTomato]; dvIs19[pAF15(gst‐4p::GFP::NLS)]	This study	N/A
*C. elegans*: AGD2937: uthIs498[bet‐1p::bet1b(CDS)::unc‐54 UTR; myo‐2p::tdTomato]; zcIs13[hsp‐6p::GFP]	This study	N/A
*C. elegans*: RHS01c: sybIS4029(bet1p::3xHA::GFP::bet‐1B)	This study	PHX4031
*C. elegans*: RHS41: uthSi7[myo‐ 3p::LifeAct::mRuby::unc‐54 3′UTR::cb‐unc‐119(+)] IV	Higuchi‐Sanabria et al. (2018)	AGD1651 4x backcross
*C. elegans*: RHS42: uthSi10[col‐ 19p::LifeAct::mRuby::unc‐54 3′UTR::cb‐unc‐119(+)] IV	Higuchi‐Sanabria et al. (2018)	AGD1654 4x backcross
*C. elegans*: RHS43: uthSi13[gly‐ 19p::LifeAct::mRuby::unc‐54 3′UTR::cb‐unc‐119(+)] IV	Higuchi‐Sanabria et al. (2018)	AGD1657 4x backcross
*C. elegans*: RHS44: uthSi10[col‐ 19p::LifeAct::mRuby::unc‐54 3′UTR::cb‐unc‐119(+)] IV; uthIs499 [bet‐1p::bet‐1b(CDS)::unc‐54 UTR; myo‐2p::tdTomato]	This study	N/A
*C. elegans*: RHS47: uthIs499[bet‐1p::bet1b(CDS)::unc‐54 UTR, myo‐2p::tdTomato]; uthSi13[gly‐19p::LifeAct::mRuby::unc‐54 3′UTR::cbunc‐119(+)] IV	This study	N/A

### 4.4 bet‐1A

ATGTCTGAGGGCAGCGGAGACCAATCACAACAACGACCATGGGCAAGTCCGCGACAGCAA

CCAATCAAAGGAATCGTACAGCCACGAGTACTTCCACCATTCGGAAAGCCAACACGACACA

CAAACAAACTGGACTACATTATGACAACAGTACTCAAAGAGGCTGGAAAACATAAACATGT

CTGGCCGTTTCAGAAGCCCGTCGATGCGGTTGCTTTATGTATTCCTCTATATCACGAGAGA

GTCGCCCGACCAATGGACTTGAAAACAATCGAGAATAGACTGAAAAGTACTTATTACACAT

GTGCTCAAGAATGCATTGATGATATCGAAACAGTTTTCCAAAACTGCTACACATTCAATGGG

AAAGAGGACGACGTGACAATTATGGCCCAAAATGTGCACGAAGTGATAAAAAAGTCACTGG

AACAAGCACCTCGCGAAGAGCATGATATGGATGTTTATTGGGGAAAAAATAAGAAAAAACC GGCAAAAAGTGACGGTGGATCGAAATCTTCGTCGAGCAAGAAGAATGATGCTCGTGGACC

ATCTGAAGCACCGTCAGAGGCTGGAAGTGAAGTTTCGTCTGTAACAACAGCATCAGCAGC AGCCCCGACGGTTTCTGAGTCTGCGAGTGTTGCCGCGAAGCCAGAACGAAAAGTGGCCG

GAAAGAAGACGGGAAAACGAAAAGCCGAATCAGAAGATGACGAGAAGCCGGAACCTTTGA GAGCAAAACGAGAGGTGGCTGTTGTCAAAAAAGAAGTTCATCAGCCATTGCTCCCAAGTAT

GAAGCCCTGTCTGAAGCTGCTCAATGATTTTTCTACAAAAAAATATCAGGAATTTGCTTGGC CATTCAACGAACCAGTAGACGCTGAACAACTGGGACTCCATGATTATCATAAAATTATCAAA

GAACCAATGGATCTGAAATCAATGAAAGCAAAAATGGAAAGTGGAGCATACAAGGAACCTT

CAGATTTCGAGCATGATGTTCGTTTAATGCTCAGGAATTGTTTTCTTTATAATCCAGTCGGT

GATCCGGTTCACAGTTTTGGTCTTAGGTTTCAAGAAGTTTTTGATAGACGATGGGCTGAACT

AGGTGATTCGAGTTCTCGTGCTTCATCAGTTGCACCTCAATCAGCTCCGATTGCTCCAACT

CCGAAAGTAGCAAAATCAAGTGCTCCAAAAGAACCGAAAGAGTCTCGAAAAGAGCATAAAA

AGGAGACGACTTTTGAAGCAAGCGGTGCAAAATCGGAGGATTTAATGCAGATAAACAACGC

GTTGAGCATGATTCGAGAACGTGAGGAAAAGCTTAAAGCAGAGCTCGCCGCTGCACAAGC GATAAAGGATAAACTGACGAGTGTGAAGAATCGACGAGAAGATAATCCGAATGAGCCATTT

CCGGAGAAGCTTATCAATGAGACAAGAGCCTTGTGCACGACGCAAGTTGGACAAAATGCTT

CAAGTTCTTCAGCTTCTTCTGCTGCTTTGAGGAACGGACGAAGCAAAAAAGCAGCATCCGC

ACGTCTCTATGGTTACGAATTTGATTCGGATGATGAGGATAATAAGATGGCACTGACTTATG

AGGAAAAACGAAACTTGAGCAATCTGATTAATAATTTACCCAACAATCAACTCAACACCATA

ATTTCGATTATTCAACGGAGAGAACGAAGCGCTCTGATGCAACAACAACTCGATGACAGTG

AGGTTGAACTGGATTTCGAATCACTTGGAGATATGTGCCTGAGAGAAATGGGTGCATTTAT

CAAAACAATTCCAACATTAAACGGAAATGGCGATGATGAGAAGCCGAAAACGTCTTCGAATCCGACATCTTCTGGAGCAACAGGATCAAAGGGTTCGTCGTCGTTGGAGAGCAAAAATGGA AAGAAAAAGAAAAACTTCAATATGTCCGAATCCTCGGATGATGAGACGTCGAATAGTCGAA

AACGTCGAAAGAGAGAGAGCAGTGAATCACAGAGCTCTTCGTCCAGTGATGATGATTCAGA

TGATGAGGATAGGCCGAGTATTCCCCGTAAATCAGGTCAACCACCATCAACATCACGTGAA

TGGAATCAATCATCAGCTCCTCCACCACGAATGGGAGGAATGGGAGGACAACCACCAATG

TCACGAGTACCTGCATCATCATCCACATCTGTATCAGCAATCGGAAAGAACAACGCAGCCG

CCTCGTCGAATTCATATCAAGCTCCAAAACCTGCACCAGTACCAGCACCAACATCATCAAG

ACCTCCGGCAGCACCGAGACCACCGTCAAAACCAAAGAAAACGGGTGGAGCGAGTATTCT

TGATACTCTACTTCCAGATACATTTGGAGCATCACCTCCCCAGTTTTTCCAGTCGCAACCAA

CAACGTCGGCTACGATTAGATCACCAACGGAAAGCCAACCCGGGAATGGTGAAGACGAGC

AGACCAGGATTCAGAGGATGCGGATGGAGGCAAAGCGAGCCCGCCAAAAAGAAGACGAA

GGCAGTGTCTCGTTGTCAAACCAAATGGAAATGATGGCTGCATTTGAATTTGATAATACATA

TTAA

### 4.5 bet‐1B

ATGTCTGAGGGCAGCGGAGACCAATCACAACAACGACCATGGGCAAGTCCGCGACAGCAA

CCAATCAAAGGAATCGTACAGCCACGAGTACTTCCACCATTCGGAAAGCCAACACGACACA

CAAACAAACTGGACTACATTATGACAACAGTACTCAAAGAGGCTGGAAAACATAAACATGT

CTGGCCGTTTCAGAAGCCCGTCGATGCGGTTGCTTTATGTATTCCTCTATATCACGAGAGA

GTCGCCCGACCAATGGACTTGAAAACAATCGAGAATAGACTGAAAAGTACTTATTACACAT

GTGCTCAAGAATGCATTGATGATATCGAAACAGTTTTCCAAAACTGCTACACATTCAATGGG

AAAGAGGACGACGTGACAATTATGGCCCAAAATGTGCACGAAGTGATAAAAAAGTCACTGG

AACAAGCACCTCGCGAAGAGCATGATATGGATGTTTATTGGGGAAAAAATAAGAAAAAACC GGCAAAAAGTGACGGTGGATCAAATCTTCGTCGAGCAAGAAGAATGATGCTCGTGGACC

ATCTGAAGCACCGTCAGAGGCTGGAAGTGAAGTTTCGTCTGTAACAACAGCATCAGCAGC AGCCCCGACGGTTTCTGAGTCTGCGAGTGTTGCCGCGAAGCCAGAACGAAAAGTGGCCG

GAAAGAAGACGGGAAAACGAAAAGCCGAATCAGAAGATGACGAGAAGCCGGAACCTTTGA GAGCAAAACGAGAGGTGGCTGTTGTCAAAAAAGAAGTTCATCAGCCATTGCTCCCAAGTAT

GAAGCCCTGTCTGAAGCTGCTCAATGATTTTTCTACAAAAAAATATCAGGAATTTGCTTGGC

CATTCAACGAACCAGTAGACGCTGAACAACTGGGACTCCATGATTATCATAAAATTATCAAA

GAACCAATGGATCTGAAATCAATGAAAGCAAAAATGGAAAGTGGAGCATACAAGGAACCTT

CAGATTTCGAGCATGATGTTCGTTTAATGCTCAGGAATTGTTTTCTTTATAATCCAGTCGGT

GATCCGGTTCACAGTTTTGGTCTTAGGTTTCAAGAAGTTTTTGATAGACGATGGGCTGAACT

AGGTGATTCGAGTTCTCGTGCTTCATCAGTTGCACCTCAATCAGCTCCGATTGCTCCAACT

CCGAAAGTAGCAAAATCAAGTGCTCCAAAAGAACCGAAAGAGTCTCGAAAAGAGCATAAAA

AGGAGACGACTTTTGAAGCAAGCGGTGCAAAATCGGAGGATTTAATGCAGATAAACAACGC

GTTGAGCATGATTCGAGAACGTGAGGAAAAGCTTAAAGCAGAGCTCGCCGCTGCACAAGC

GATAAAGGATAAACTGACGAGTGTGAAGAATCGACGAGAAGATAATCCGAATGAGCCATTT

CCGGAGAAGCTTATCAATGAGACAAGAGCCTTGTGCACGACGCAAGTTGGACAAAATGCTT CAAGTTCTTCAGCTTCTTCTGCTGCTTTGAGGAACGGACGAAGCAAAAAAGCAGCATCCGC

ACGTCTCTATGGTTACGAATTTGATTCGGATGATGAGGATAATAAGATGGCACTGACTTATG

AGGAAAAACGAAACTTGAGCAATCTGATTAATAATTTACCCAACAATCAACTCAACACCATA

ATTTCGATTATTCAACGGAGAGAACGAAGCGCTCTGATGCAACAACAACTCGATGACAGTG

AGGTTGAACTGGATTTCGAATCACTTGGAGATATGTGCCTGAGAGAAATGGGTGCATTTAT

CAAAACAATTCCAACATTAAACGGAAATGGCGATGATGAGAAGCCGAAAACGTCTTCGAAT CCGACATCTTCTGGAGCAACAGGATCAAAGGGTTCGTCGTCGTTGGAGAGCAAAAATGGA AAGAAAAAGAAAAACTTCAATATGTCCGAATCCTCGGATGATGAGACGTCGAATAGTCGAA

AACGTCGAAAGAGAGAGAGCAGTGAATCACAGAGCTCTTCGTCCAGTGATGATGATTCAGA

TGATGAGGATAGGCCGAGTATTCCCCGTAAATCAGGTCAACCACCATCAACATCACGTGAA

TGGAATCAATCATCAGCTCCTCCACCACGAATGGGAGGAATGGGAGGACAACCACCAATG

TCACGAGTACCTGCATCATCATCCACATCTGTATCAGCAATCGGAAAGAACAACGCAGCCG

CCTCGTCGAATTCATATCAAAAATTTTATAATTGTTTTCACAGTTATACTCCACCTTTAAAAG

TTGAAAAAAAAATCATCAAATTACTGGTAAATTTTTGTTAA *bet‐1C*

ATGTCTGAGGGCAGCGGAGACCAATCACAACAACGACCATGGGCAAGTCCGCGACAGCAA

CCAATCAAAGGAATCGTACAGCCACGAGTACTTCCACCATTCGGAAAGCCAACACGACACA

CAAACAAACTGGACTACATTATGACAACAGTACTCAAAGAGGCTGGAAAACATAAACATGT

CTGGCCGTTTCAGAAGCCCGTCGATGCGGTTGCTTTATGTATTCCTCTATATCACGAGAGA

GTCGCCCGACCAATGGACTTGAAAACAATCGAGAATAGACTGAAAAGTACTTATTACACAT

GTGCTCAAGAATGCATTGATGATATCGAAACAGTTTTCCAAAACTGCTACACATTCAATGGG

AAAGAGGACGACGTGACAATTATGGCCCAAAATGTGCACGAAGTGATAAAAAAGTCACTGG

AACAAGCACCTCGCGAAGAGCATGATATGGATGTTTATTGGGGAAAAAATAAGAAAAAACC

GGCAAAAAGTGACGGTGGATCGAAATCTTCGTCGAGCAAGAAGAATGATGCTCGTGGACC

ATCTGAAGCACCGTCAGAGGCTGGAAGTGAAGTTTCGTCTGTAACAACAGCATCAGCAGC AGCCCCGACGGTTTCTGAGTCTGCGAGTGTTGCCGCGAAGCCAGAACGAAAAGTGGCCG

GAAAGAAGACGGGAAAACGAAAAGCCGAATCAGAAGATGACGAGAAGCCGGAACCTTTGA GAGCAAAACGAGAGGTGGCTGTTGTCAAAAAAGAAGTTCATCAGCCATTGCTCCCAAGTAT GAAGCCCTGTCTGAAGCTGCTCAATGATTTTTCTACAAAAAAATATCAGGAATTTGCTTGGC

CATTCAACGAACCAGTAGACGCTGAACAACTGGGACTCCATGATTATCATAAAATTATCAAA

GAACCAATGGATCTGAAATCAATGAAAGCAAAAATGGAAAGTGGAGCATACAAGGAACCTT

CAGATTTCGAGCATGATGTTCGTTTAATGCTCAGGAATTGTTTTCTTTATAATCCAGTCGGT

GATCCGGTTCACAGTTTTGGTCTTAGGTTTCAAGAAGTTTTTGATAGACGATGGGCTGAACT

AGGTGATTCGAGTTCTCGTGCTTCATCAGTTGCACCTCAATCAGCTCCGATTGCTCCAACT

CCGAAAGTAGCAAAATCAAGTGCTCCAAAAGAACCGAAAGAGTCTCGAAAAGAGCATAAAA

AGGAGACGACTTTTGAAGCAAGCGGTGCAAAATCGGAGGATTTAATGCAGATAAACAACGC

GTTGAGCATGATTCGAGAACGTGAGGAAAAGCTTAAAGCAGAGCTCGCCGCTGCACAAGC

GATAAAGGATAAACTGACGAGTGTGAAGAATCGACGAGAAGATAATCCGAATGAGCCATTT

CCGGAGAAGCTTATCAATGAGACAAGAGCCTTGTGCACGACGCAAGTTGGACAAAATGCTT

CAAGTTCTTCAGCTTCTTCTGCTGCTTTGAGGAACGGACGAAGCAAAAAAGCAGCATCCGC

ACGTCTCTATGGTTACGAATTTGATTCGGATGATGAGGATAATAAGATGGCACTGACTTATG

AGGAAAAACGAAACTTGAGCAATCTGATTAATAATTTACCCAACAATCAACTCAACACCATA

ATTTCGATTATTCAACGGAGAGAACGAAGCGCTCTGATGCAACAACAACTCGATGACAGTG

AGGTTGAACTGGATTTCGAATCACTTGGAGATATGTGCCTGAGAGAAATGGGTGCATTTAT

CAAAACAATTCCAACATTAAACGGAAATGGCGATGATGAGAAGCCGAAAACGTCTTCGAAT CCGACATCTTCTGGAGCAACAGGATCAAAGGGTTCGTCGTCGTTGGAGAGCAAAAATGGA

AAGAAAATAA

### 4.6 bet‐1p

CACAGGTCTCTAGTGTATCCACTTCGAATGCGATGCCCGAAACCTCTTCATCCATCCGTCT

CCTTCTCGCTCTCTCTCTCTCTCTCTCTTCTCCATCTCTCTCCACATTTTGCCTGCTATCTCG

TGATTGTCGTCCCGTCCGTGTTCCGCCGCACACACTGCCTGTCTTCTCTTAACCGTGTGTC

GATCAACTCCCAAACCGCTACGCTATTTCTCTCTCCCTCTCTCTCTCTCTTCGGCGGTGAC

ATTTCTGACTAGATGGTCATACAAAACGCGTGCTGCGCGCGCGCTCCGCAAAAATCGACG

CGAATCGATTAATGTGCGTCTCGTTTCTCTATCTCTGACCGCCCCCGCTTCAACCTAACACT

ATTTTTGAATGCTTTTCAACTGTAACTTGCAGCTAATTAGAAGTTGAGAGATAACCTGTTGC

GATTGGCTCCGGGCAAGGGTTGGGAGGTCGCACCAGAAATTTTAGAGCTCTAGGATTTCA

AATTTTTGGGTTTCAAGACCGTAACATGATTTTCTTGGAAATTTATCACAAATCATGTAGAAA

ATCGATATCAGTAAGAGGGAGTGAGTGATCTATCATTTTTTATCTTTCGATCTGAAATTCCA

CAGCGAAGGTTTTCTGCCGAAATTTCGAAATTGGTATTTTGAACTATCCGATAATTCGTAGA

ACATCAAGATAAAGTGTCAACCTATAGAAAATCACATGATTCGTCAGAAAATAACATTAATTT

CATATGAAATAGTTGAGAAAGTGCTCAAAAATGGCCTAAAATTATCCAATAATCGACATTTG

ACAACTTTCAGCACACTTTTGAACCGTTTATCAATTGTTTCTGCTGAAATAGACGTATTTTTC

GGACGAATCGAGTGATTTCCTATAGTTTTACACTGATTTTTGACAAAAAAATATTGATAGAAC

ATGGTGCATTAGGCAATTTTTTAGAATTGCCGTCTACACCTGATTTCGATGGGTCCTCGTGA

CAAGACCCAAAATTTTATTATTTTTATCGTTGAAAAAAATCAAATCAATAACACCGCAATCAC CATTTGCAAAGTTTAATTAAATACAATTTTTATTAAAATATTTCAGGAATAAAAATATTAGTCA

GAATAATCCCATGTTCTTCTAGCGATTTCAACTAATTCTTTGAAAATAACATTTCTTGGAATT TAAGAATACGAAAATAGTCACTTCTTTGTATTCTAGAAACGCTAATTCCTGCAACCGACAAA TTAAAAGTACAAAAAATGATACGGCAAGCGCGCTCCAATTCAAATCGAGTCTCCCGCCTTC

CTTGACGTCATTGCTAACAGCTGCTTCGGTTTTTTCCTCCAAATTTCGTGGTTCAAATTTTAT TTTTAATTGAATTTTAACAAAATAGGAAGCTAGTTGAGTAACATTTATTATTAATTTTGTAAAA

TATTCTGCAAATTCGGCGTTTTCTTTTAATTCAAATAAAAGTTTTCAATAAAAAAAATCGATAT

TTTCAG

### 4.7 unc‐54 3′ UTR

CATCTCGCGCCCGTGCCTCTGACTTCTAAGTCCAATTACTCTTCAACATCCCTACATGCTCT

TTCTCCCTGTGCTCCCACCCCCTATTTTTGTTATTATCAAAAAACTTCTCTTAATTTCTTTGTT

TTTTAGCTTCTTTTAAGTCACCTCTAACAATGAAATTGTGTAGATTCAAAAATAGAATTAATT

CGTAATAAAAAGTCGAAAAAAATTGTGCTCCCTCCCCCCATTAATAATAATTCTATCCCAAA

ATCTACACAATGTTCTGTGTACACTTCTTATGTTTTTTACTTCTGATAAATTTTTTTGAAACAT

CATAGAAAAAACCGCACACAAAATACCTTATCATATGTTACGTTTCAGTTTATGACCGCAAT

TTTTATTTCTTCGCACGTCTGGGCCTCTCATGACGTCAAATCATGCTCATCGTGAAAAAGTT

TTGGAGTATTTTTGGAATTTTTCAATCAAGTGAAAGTTTATGAAATTAATTTTCCTGCTTTTG

CTTTTTGGGGTTTCCCCTATTGTTTGTCAAGATTTCGAGGACGGCGTTTTTCTTGCTAAAAT

CACAAGTATTGATGAGCACGATGCAAGAAAGATCGGAAGAAGGTTTGGGTTTGAGGCTCA GTGGAAG

### 4.8 GFP used in *GFP*::*3xHA*::*bet‐1* construct (introns are lower case, stop codon removed)

ATGAGTAAAGGAGAAGAACTTTTCACTGGAGTTGTCCCAATTCTTGTTGAATTAGATGGTGA

TGTTAATGGGCACAAATTTTCTGTCAGTGGAGAGGGTGAAGGTGATGCAACATACGGAAAA

CTTACCCTTAAATTTATTTGCACTACTGGAAAACTACCTGTTCCATGGgtaagtttaaacatatatatac taactaaccctgattatttaaattttcagCCAACACTTGTCACTACTTTCTGTTATGGTGTTCAATGCTTCTC

GAGATACCCAGATCATATGAAACGGCATGACTTTTTCAAGAGTGCCATGCCCGAAGGTTAT GTACAGGAAAGAACTATATTTTTCAAAGATGACGGGAACTACAAGACACgtaagtttaaacagttcgg tactaactaaccatacatatttaaattttcagGTGCTGAAGTCAAGTTTGAAGGTGATACCCTTGTTAATAGA ATCGAGTTAAAAGGTATTGATTTTAAAGAAGATGGAAACATTCTTGGACACAAATTGGAATA CAACTATAACTCACACAATGTATACATCATGGCAGACAAACAAAAGAATGGAATCAAAGTTgt

aagtttaaacatgattttactaactaactaatctgatttaaattttcagAACTTCAAAATTAGACACAACATTGAAGATG

GAAGCGTTCAACTAGCAGACCATTATCAACAAAATACTCCAATTGGCGATGGCCCTGTCCT

TTTACCAGACAACCATTACCTGTCCACACAATCTGCCCTTTCGAAAGATCCCAACGAAAAGA

GAGACCACATGGTCCTTCTTGAGTTTGTAACAGCTGCTGGGATTACACATGGCATGGATGA

ACTATACAAA

### 4.9 3xHA

TATCCATATGACGTGCCGGACTACGCGTACCCGTATGATGTTCCAGACTACGCCTATCCGT

ACGACGTACCAGATTATGCA

### 4.10 *bet‐1* RNAi

CAGCAACCAATCAAAGGAATCGTACAGCCACGAGTACTTCCACCATTCGGAAAGCCAACAC

GACACACAAACAAACTGGACTACATTATGACAACAGTACTCAAAGAGGCTGGAAAACATAA

ACATGTCTGGCCGTTTCAGAAGCCCGTCGATGCGGTTGCTTTATGTATTCCTCTATATCAC

GAGAGAGTCGCCCGACCAATGGACTTGAAAACAATCGAGAATAGACTGAAAAGTACTTATT

ACACATGCGCTCAAGAATGCATTGATGATATCGAAACAGTTTTCCAAAACTGCTACACATTC

AATGGGAAAGAGGACGACGTGACAATTATGGCCCAAAATGTGCACGAAGTGATAAAAAAGT

CACTGGAACAAGCACCTCGCGAAGAGCATGATATGGATGTTTATTGGGGAAAAAATAAGAA

AAAACCGGCAAAAAGTGACGGTGGATCGAAATCTTCGTCGAGCAAGAAGAATGATGCTCG

TGGACCATCTGAAGCACCGTCAGAGGCTGGAAGTGAAGTTTCGTCTGTAACAACAGCATCA

GCAGCAGCCCCGACGGTTTCTGAGTCTGCGAGTGTTGCCG

### 4.11 *mys‐1* RNAi

AAAAAAGCAGGCTTGACCGAGCCGAAGAAGGAGATTATAGAGGACGAAAATCATGGAATAT CCAAGAAAATACCAACAGATCCCAGGCAATACGAGAAAGTTACAGAGGGATGCCGGTTATT

GGTCATGATGGCTTCACAAGAAGAAGAAAGATGGGCCGAAGTTATTTCAAGATGCCGAGCT

GCAAATGGTTCAATTAAATTCTATGTCCATTATATCGATTGCAACCGAAGACTTGACGAATG

GGTTCAGTCTGATAGGCTCAATTTAGCGTCGTGTGAGCTACCAAAAAAAGGAGGAAAGAAA

GGAGCACACTTGCGGGAAGAAAATCGAGATTCGAATGAAAATGAAGGAAAGAAAAGCGGC

CGAAAACGAAAGATTCCACTACTTCCGATGGATGATCTCAAGGCGGAATCCGTAGATCCAT

TACAAGCAATTTCAACGATGACCAGCGGATCTACTCCAAGTCTTCGAGGTTCCATGTCGAT

GGTCGGCCATAGTGAAGATGCAATGACAAGGATCCGAAATGTCGAATGCATTGAACTAGG

AAGATCACGAATTCAGCCATGGTACTTTGCACCTTATCCACAACAATTGACAAGTTTGGATT

GTATTTATATTTGCGAATTTTGTCTGAAATATCTAAAGTCGAAAACTTGTCTGAAACGGCAC

NTGGAAAAATGTGCAATGTGTCACCCACCTGGCAATCAAATCTACAGTCACGATAAACTTTC

ATTTTTTGAAATCGACGGCCGCAAAAACAAAAGCTATGCTCAGAATCTATGCCTGCTTGCCA AACTT

### 4.12 *Caenorhabditis elegans* screen

*C. elegans* orthologs of human genes from the cytochalasin screen were identified using Ortholist 2 (Kim et al., 2018). RNAis were isolated from the Vidal (Reboul et al., 2001) or Ahringer (Lee et al., 2003) RNAi libraries and sequence‐verified using standard sanger sequencing. RNAi constructs that matched the expected sequences at a bp length > 150 were included in the screen. For synthetic lethality screens, RNAi cultures were grown in a deep‐well 96‐well plate to saturation and were mixed at a 10%/90% ratio of *act‐1* RNAi to candidate gene RNAi. Animals were grown on these 10%/90% mix and grown at 20°C and screened at day 1 of adulthood. All hits are defined as those that show any observable difference between the 10%/90% *act‐1*/gene mix in comparison to 100% candidate gene RNAi alone. All screening was performed with the researcher blind to the identity of each RNAi and was screened by two independent researchers. Only hits that had phenotypes scored as positive by both researchers were included as hits and images are made available in Figure 1.

### 4.13 *Caenorhabditis elegans* microscopy

Animals were always imaged at the specified ages in figure legends using standard bleaching protocols for synchronization. For all aging experiments, animals were aged on RNAi plates supplemented with FUDR from day 1 of adulthood until the desired stage. 100 μl of 10 mg/ml FUDR were spotted on the bacterial lawn. At least 1 replicate was performed without FUDR and manual picking of animals away from their progeny as previously described (Higuchi‐Sanabria et al., 2018) to ensure that measurable effects were independent of FUDR. For all microscopy, representative images of three independent biological replicates are shown.

For high magnification live‐cell imaging, animals are picked off of plates and mounted directly onto a microscope slide containing M9 + 0.1 M sodium azide. For standard wide‐field microscopy (*myo‐3p*::*LifeAct* and *col‐19p*::*LifeAct*), images were acquired on either a Zeiss AxioObserver.Z1 microscope equipped with a lumencor sola light engine and Ziess axiocam 506 camera driven by Zeiss ZenBlue software using a 63x/1.4 PlanApochromat objective, standard dSRed filter (Zeiss filter set 43), and a DFC9000 camera; or a Leica Thunder Imager equipped with a 63x/1.4 Plan AproChromat objective, standard dsRed filter (11525309), Leica DFC9000 GT camera, a Leica LED5 light source, and run on LAS X software. For confocal microscopy (*gly‐19p*::*LifeAct*), imaging was performed on a Stellaris 5 confocal microscope equipped with a white light laser source and spectral filters, HyD detectors, 63x/1.4 Plan ApoChromat objective, and run on LAS X software.

For imaging of GFP::BET‐1, animals were bleached to isolate eggs. 100 μl of egg/M9 mix was mixed with 500 μl of 4% PFA diluted in PBS and fixed at room temperature on an orbital shaker for 11 min. Samples were frozen at −80°C until imaging. Prior to imaging, PFA was washed using 1 ml of PBS and shaking on an orbital shaker for 10 min at room temperature. A total of 3x PBS washes was performed. For staining, samples were submerged in 1 ml of PBS, then 0.75 μl of 5 mg/ml DAPI dissolved in DMSO was added. Eggs were incubated with DAPI for 50 min at room temperature on an orbital shaker. Excess DAPI was washed with 3x PBS washes at 10 min each at room temperature on the orbital shaker. 5 μl of egg/PBS mix was mounted onto a slide and mixed with 5 μl of VectaShield mounting media and imaged on a Stellaris 5 confocal microscope equipped with a white light laser source, HyD detector, 63x/1.4 Plan ApoChromat objective, and run on LAS X software.

For imaging of fluorescent transcriptional reporters, animals were synchronized via bleaching and grown on standard RNAi plates and imaged at day 1 of adulthood. For imaging, animals were moved onto standard NGM plates without bacteria containing 5 μl of 100 mM sodium azide to paralyze worms. Paralyzed worms were lined up and imaged immediately on a Leica M205FCA automated fluorescent stereoscope equipped with a standard GFP filter and Leica K5 camera and run on LAS X software. 3 biological replicates were performed per experiment with *n* > 12 per replicate. All detailed protocols are available at (Bar‐Ziv et al., 2020), but also briefly described: For *hsp‐6p*::*GFP*, animals were grown on RNAi plates until day 1 and imaged immediately. For *gst‐4p*::*GFP*, animals were grown on RNAi plates until the L4 stage, washed off plates with M9, treated with 1 mM tertbutyl hydroperoxide for 4 h (or equivalent water for controls) floating in M9 in a rotator at 20°C, then washed 3x with M9 and plated on standard OP50 plates for 24 h, then imaged.

### 4.14 Gut bacteria invasion assay

To measure gut bacteria invasion, animals were synchronized via bleaching and plated from hatch on RNAi of choice mixed with 20% HT115 bacteria expressing mCherry as previously described (Egge et al., 2019). At the desired age, animals are transferred by hand onto a standard OP50 plate and allowed to feed on OP50 for 2 h at 20°C to clarify excess mCherry bacteria. For imaging, animals are lined up on a standard NGM plate without bacteria in M9 + 0.1 M sodium azide. Images were acquired on a Leica M205FCA equipped with a standard dsRed filter and Leica K5 camera and run on LAS X software. For each of 3 biological replicates, 2 technical replicates of 12+ animals per replicate were imaged. For each technical and biological replicate, the % of animals with bacterial invasion were quantified and plotted. Statistical analysis was performed across all replicates using a standard *t*‐test using Prism software.

### 4.15 *Caenorhabditis elegans* RT‐PCR and RNA‐seq analysis

For RNA isolation, all RNA collection was performed at day 1 of adulthood. ~1000 animals were harvested from RNAi plates using M9. Animals were pelleted by gravity by allowing adult worms to settle to the bottom of the tube and aspirating off eggs and L1. Animals were washed and gravity settled 3x to remove a majority of progeny, then animals were placed into Trizol solution and worms were freeze/thawed 3x with liquid nitrogen with a 30 sec vortexing step between each freeze cycle. After the final thaw, chloroform was added at a 1:5 chlorform/trizol ratio and aqueous separation of RNA was performed via centrifugation in a heavy gel phase‐lock tube (VWR, 10847–802). The aqueous phase was mixed 1:1 with isopropanol then applied to a Qiagen RNeasy Mini Kit (74106) and RNA purification was performed as per manufacturer's directions.

Library preparation was performed using a Kapa Biosystems mRNA Hyper Prep Kit sequencing was performed at the Vincent J Coates Genomic Sequencing Core at the University of California, Berkeley using an Illumina HS4000 mode SR100. Four biological replicates were measured per condition. Reads per gene were quantified using kallisto (Bray et al., 2016), with WBcel235 as the worm reference genome. Fold changes were determined using DESeq2 (Love et al., 2014). XBP‐1 gene targets were defined as previously experimentally determined (Urano et al., 2002). GO enrichment was calculated using WormEnrichr (Chen et al., 2013). *bet‐1B* overexpression, *bet‐1* RNAi, and *bet‐1(uth41)* mutants were compared to N2 wild‐type control. In addition, *bet‐1B* overexpressing worms were grown on *mys‐1* RNAi and compared to a *mys‐1* RNAi control.

For RT‐PCR, cDNA synthesis was performed using the QuantaBio cDNA supermix Qscript (101414‐102) using 1 μg of RNA. RT‐PCR was performed using NEB Q5 DNA polymerase as per manufacturer's guidelines using primers listed below. Four biological replicates were performed per condition. Image quantification was performed using ImageJ by drawing an ROI 974 of equal size around each band and quantifying for integrated density. Data was normalized to a 975 *tba‐1* loading control.

**Table:** 

Primers used in this study		
Primer name	Primer sequence	Primer purpose
bet‐1 qPCR F	CCAACCCGGGAAT GGTGAAGAC	Forward primer to RT‐PCR/qPCR *bet‐1* without overlapping with RNAi sequence
bet‐1 qPCR R	CCATCATTTCCATT TGGTTTGACAACG	Reverse primer to RT‐PCR/qPCR *bet‐1* without overlapping with RNAi sequence
mys‐1 qPCR F	CAGATCATGTTCT AGCAACAACG	Forward primer to RT‐PCR/qPCR *mys‐1* without overlapping with RNAi sequence
mys‐1 qPCR R	GATAGCGTAAGCT TTTCGGTG	Reverse primer to RT‐PCR/qPCR *mys‐1* without overlapping with RNAi sequence
tbas‐1 qPCR F	TCAACACTGCCAT CGCCGCC	Forward primer to RT‐PCR/qPCR *tba‐1* loading control
tba‐1 qPCR R	TCCAAGCGAGACC AGGCTTCAG	Reverse primer to RT‐PCR/qPCR *tba‐1* loading control

### 4.16 *Caenorhabditis elegans* thrashing assay

Thrashing assays were performed on animals synchronized via bleaching and aged on plates containing FUDR from day 1. 100 μl of 10 mg/ml FUDR were spotted on the bacterial lawn. At the desired age, plates containing adult animals were flooded with 100 μl of M9 solution, and 30 sec videos were acquired on an M205FCA stereomicroscope equipped with a Leica K5 microscope and run on LAS X software. Thrashing was measured by eye over a 10 s period. A single trash is defined as bending of >50% of the animal's body in the opposite direction. Representative data of three independent biological replicates are presented. Dot plots were generated using Prism 7 software where every dot represents a single animal and lines represent median and interquartile range. All statistics were performed using nonparametric Mann–Whitney testing.

### 4.17 *Caenorhabditis elegans* brood size assay

A synchronized population of animals were collected via bleaching and 10 L4 animals were moved onto individual plates. Every 12 h, animals were moved onto fresh plates and plates containing eggs were stored in a 15°C incubator for 2–3 days. All live progeny on every egg‐lay plate were scored and summed to determine brood size. Dot plots were generated using Prism 7 software where every dot represents a single animal and lines represent median and interquartile range. All statistics were performed using non‐parametric Mann–Whitney testing.

### 4.18 *Caenorhabditis elegans* life span assay

*Caenorhabditis elegans* life span assays were performed on standard RNAi plates and were all performed at 20°C as previously described (Bar‐Ziv et al., 2020). Adult worms were moved away from progeny daily onto fresh RNAi plates until no progeny were visible (~7–8 days). Animals were then scored every other day until all animals were scored as either dead or censored. All animals exhibiting bagging, intestinal leaking out of the vulva, or other age‐unrelated death were censored and removed from quantification. For life spans on FUDR, animals were grown on RNAi plates supplemented with FUDR from day 1 of adulthood until the desired stage. 100 μl of 10 mg/ml FUDR were spotted on the bacterial lawn. At least 1 replicate for every life span was performed in the absence of FUDR. For tunicamycin and paraquat survival assays, animals are grown on RNAi plates until day 1 of adulthood. At day 1, animals are moved onto RNAi plates containing 25 μg/ml tunicamycin or 2.5 mM paraquat as previously described (Castro Torres et al., 2022). All statistical analysis was performed using Prism7 software using LogRank testing. All life span experiments were performed with researchers blinded to sample conditions. Representative data are represented in figures and all replicates are made available in Tables S6 and S7.

## AUTHOR CONTRIBUTIONS

G.G. and R.H.S. designed all experiments, performed or oversaw all experiments, and prepared the figures and manuscript. R.B.Z. performed computational analysis, figure construction, and writing for all transcriptomics data. M.A. performed all *Caenorhabditis elegans* experiments for manuscript revision. N.Dasgupta performed all senescent cell culture experiments. N.Dutta performed RTPCR analysis and assisted with life spans. H.Z., and W.F. performed all standard cell culture experiments. C.K.T., E.A.M., and O.S. performed all computational analysis for CRISPR‐Cas9 screening. D.M., A.A., S.H., and T.C.T. assisted with *C. elegans* experiments. P.D.A. supervised all senescent cell culture experiments. M.A.T. performed essential experiments that assisted in development of the manuscript. All authors edited the manuscript.

## CONFLICT OF INTEREST

All authors of the manuscript declare that they have no competing interests.

## Supporting information


Figures S1–S9

Table S1

Table S2

Table S3

Table S4

Table S5

Table S6

Table S7

Table S8

## Data Availability

All data required to evaluate the conclusions in this manuscript are available within the manuscript and Supplementary Materials. All strains synthesized in this manuscript are derivatives of N2 or other strains from CGC and are either made available on CGC or available upon request. All raw datasets, including CRISPR‐Cas9 and RNA‐seq, are available through Annotare 2.0 Array Express Accession E‐MTAB‐11786 and E‐MTAB‐12289.

## References

[acel13742-bib-0001] Acsadi, G. , Dickson, G. , Love, D. R. , Jani, A. , Walsh, F. S. , Gurusinghe, A. , Wolff, J. A. , & Davies, K. E. (1991). Human dystrophin expression in mdx mice after intramuscular injection of DNA constructs. Nature, 352, 815–818. 1881437 10.1038/352815a0

[acel13742-bib-0002] Alim, M. A. , Hossain, M. S. , Arima, K. , Takeda, K. , Izumiyama, Y. , Nakamura, M. , Kaji, H. , Shinoda, T. , Hisanaga, S. , & Ueda, K. (2002). Tubulin seeds alpha‐synuclein fibril formation. The Journal of Biological Chemistry, 277, 2112–2117. 11698390 10.1074/jbc.M102981200

[acel13742-bib-0003] Alim, M. A. , Ma, Q.‐L. , Takeda, K. , Aizawa, T. , Matsubara, M. , Nakamura, M. , Asada, A. , Saito, T. , Kaji, H. , Yoshii, M. , Hisanaga, S. , & Uéda, K. (2004). Demonstration of a role for alpha‐synuclein as a functional microtubule‐associated protein. Journal of Alzheimer's Disease, 6, 435–442; discussion 443–449. 10.3233/jad-2004-6412 15345814

[acel13742-bib-0004] Baird, N. A. , Douglas, P. M. , Simic, M. S. , Grant, A. R. , Moresco, J. J. , Wolff, S. C. , Yates, J. R. , Manning, G. , & Dillin, A. (2014). HSF‐1‐mediated cytoskeletal integrity determines thermotolerance and life span. Science, 346, 360–363. 25324391 10.1126/science.1253168 PMC4403873

[acel13742-bib-0005] Balch, W. E. , Morimoto, R. I. , Dillin, A. , & Kelly, J. W. (2008). Adapting proteostasis for disease intervention. Science, 319, 916–919. 18276881 10.1126/science.1141448

[acel13742-bib-0006] Bar‐Ziv, R. , Frakes, A. E. , Higuchi‐Sanabria, R. , Bolas, T. , Frankino, P. A. , Gildea, H. K. , Metcalf, M. G. , & Dillin, A. (2020). Measurements of physiological stress responses in *C. elegans* . Journal of Visualized Experiments, 159, e61001. 10.3791/61001 PMC7840273 32510480

[acel13742-bib-0007] Bear, J. E. , Svitkina, T. M. , Krause, M. , Schafer, D. A. , Loureiro, J. J. , Strasser, G. A. , Maly, I. V. , Chaga, O. Y. , Cooper, J. A. , Borisy, G. G. , & Gertler, F. B. (2002). Antagonism between Ena/VASP proteins and Actin filament capping regulates fibroblast motility. Cell, 109, 509–521. 12086607 10.1016/s0092-8674(02)00731-6

[acel13742-bib-0008] Birnbaum, A. , Wu, X. , Tatar, M. , Liu, N. , & Bai, H. (2019). Age‐dependent changes in transcription factor FOXO targeting in female drosophila. Frontiers in Genetics, 10, 312. 31134124 10.3389/fgene.2019.00312 PMC6514159

[acel13742-bib-0009] Blanpied, T. A. , Scott, D. B. , & Ehlers, M. D. (2003). Age‐related regulation of dendritic endocytosis associated with altered clathrin dynamics. Neurobiology of Aging, 24, 1095–1104. 14643381 10.1016/j.neurobiolaging.2003.04.004

[acel13742-bib-0010] Bray, N. L. , Pimentel, H. , Melsted, P. , & Pachter, L. (2016). Near‐optimal probabilistic RNA‐seq quantification. Nature Biotechnology, 34, 525–527. 10.1038/nbt.3519 27043002

[acel13742-bib-0011] Castello, P. R. , Drechsel, D. A. , & Patel, M. (2007). Mitochondria are a major source of paraquatinduced reactive oxygen species production in the brain. The Journal of Biological Chemistry, 282, 14186–14193. 17389593 10.1074/jbc.M700827200 PMC3088512

[acel13742-bib-0012] Castro Torres, T. , Moaddeli, D. , Averbukh, M. , Coakley, A. J. , Dutta, N. , Garcia, G. , & Higuchi‐Sanabria, R. (2022). Surveying low‐cost methods to measure lLife span and Healthspan in *Caenorhabditis elegans* . Journal of Visualized Experiments, 183. 10.3791/64091 PMC9881476 35665741

[acel13742-bib-0013] Caviston, J. P. , & Holzbaur, E. L. F. (2006). Microtubule motors at the intersection of trafficking and transport. Trends in Cell Biology, 16, 530–537. 16938456 10.1016/j.tcb.2006.08.002

[acel13742-bib-0014] Ceol, C. J. , & Horvitz, H. R. (2004). A new class of C. elegans synMuv genes implicates a Tip60/NuA4‐like HAT complex as a negative regulator of Ras signaling. Developmental Cell, 6, 563–576. 15068795 10.1016/s1534-5807(04)00065-6

[acel13742-bib-0015] Chakrabarti, R. , Ji, W.‐K. , Stan, R. V. , de Juan, S. J. , Ryan, T. A. , & Higgs, H. N. (2018). INF2‐Mediated Actin polymerization at the ER stimulates mitochondrial calcium uptake, inner membrane constriction, and division. The Journal of Cell Biology, 217, 251–268. 29142021 10.1083/jcb.201709111 PMC5748994

[acel13742-bib-0016] Chen, E. Y. , Tan, C. M. , Kou, Y. , Duan, Q. , Wang, Z. , Meirelles, G. V. , Clark, N. R. , & Ma'ayan, A. (2013). Enrichr: Interactive and collaborative HTML5 gene list enrichment analysis tool. BMC Bioinformatics, 14, 128. 23586463 10.1186/1471-2105-14-128 PMC3637064

[acel13742-bib-0017] Chiang, C.‐M. (2009). Brd4 engagement from chromatin targeting to transcriptional regulation: Selective contact with acetylated histone H3 and H4. F1000 Biology Reports, 1, 98. 20495683 10.3410/B1-98 PMC2873783

[acel13742-bib-0018] Cooper, G. M. (2000). Actin, myosin, and cell movement. In The cell: A molecular approach (2nd ed.). Sinauer Associates.

[acel13742-bib-0019] Costa, M. , Draper, B. W. , & Priess, J. R. (1997). The role of Actin filaments in patterning the Caenorhabditis elegans cuticle. Developmental Biology, 184, 373–384. 9133443 10.1006/dbio.1997.8530

[acel13742-bib-0020] Daniele, J. R. , Higuchi‐Sanabria, R. , Durieux, J. , Monshietehadi, S. , Ramachandran, V. , Tronnes, S. U. , Kelet, N. , Sanchez, M. , Metcalf, M. G. , Garcia, G. , Frankino, P. A. , Benitez, C. , Zeng, M. , Esping, D. J. , Joe, L. , & Dillin, A. (2020). UPRER promotes lipophagy independent of chaperones to extend life span. Science Advances, 6, eaaz1441. 31911951 10.1126/sciadv.aaz1441 PMC6938708

[acel13742-bib-0021] Davidson, A. J. , & Wood, W. (2016). Unravelling the Actin cytoskeleton: A new competitive edge? Trends in Cell Biology, 26, 569–576. 27133808 10.1016/j.tcb.2016.04.001 PMC4961066

[acel13742-bib-0022] Devaiah, B. N. , Case‐Borden, C. , Gegonne, A. , Hsu, C. H. , Chen, Q. , Meerzaman, D. , Dey, A. , Ozato, K. , & Singer, D. S. (2016). BRD4 is a histone acetyltransferase that evicts nucleosomes from chromatin. Nature Structural & Molecular Biology, 23, 540–548. 10.1038/nsmb.3228 PMC4899182 27159561

[acel13742-bib-0023] Dey, A. , Ellenberg, J. , Farina, A. , Coleman, A. E. , Maruyama, T. , Sciortino, S. , Lippincott‐Schwartz, J. , & Ozato, K. (2000). A Bromodomain protein, MCAP, associates with mitotic chromosomes and affects G2‐to‐M transition. Molecular and Cellular Biology, 20, 6537–6549. 10938129 10.1128/mcb.20.17.6537-6549.2000 PMC86127

[acel13742-bib-0024] Doench, J. G. , Fusi, N. , Sullender, M. , Hegde, M. , Vaimberg, E. W. , Donovan, K. F. , Smith, I. , Tothova, Z. , Wilen, C. , Orchard, R. , Virgin, H. W. , Listgarten, J. , & Root, D. E. (2016). Optimized sgRNA design to maximize activity and minimize off‐target effects of CRISPR‐Cas9. Nature Biotechnology, 34, 184–191. 10.1038/nbt.3437 PMC4744125 26780180

[acel13742-bib-0025] Donati, B. , Lorenzini, E. , & Ciarrocchi, A. (2018). BRD4 and cancer: Going beyond transcriptional regulation. Molecular Cancer, 17, 164. 30466442 10.1186/s12943-018-0915-9 PMC6251205

[acel13742-bib-0026] Douglas, P. M. , Baird, N. A. , Simic, M. S. , Uhlein, S. , McCormick, M. A. , Wolff, S. C. , Kennedy, B. K. , & Dillin, A. (2015). Heterotypic signals from neural HSF‐1 separate thermotolerance from longevity. Cell Reports, 12, 1196–1204. 26257177 10.1016/j.celrep.2015.07.026 PMC4889220

[acel13742-bib-0027] Duclot, F. , & Kabbaj, M. (2017). The role of early growth response 1 (EGR1) in brain plasticity and neuropsychiatric disorders. Frontiers in Behavioral Neuroscience, 11, 35. 10.3389/fnbeh.2017.00035 28321184 PMC5337695

[acel13742-bib-0028] Dutta, N. , Garcia, G. , & Higuchi‐Sanabria, R. (2022). Hijacking cellular stress responses to promote lLife span. Frontiers in Aging, 3, 860404. 10.3389/fragi.2022.860404 35821861 PMC9261414

[acel13742-bib-0029] Egge, N. , Arneaud, S. L. B. , Wales, P. , Mihelakis, M. , McClendon, J. , Fonseca, R. S. , Savelle, C. , Gonzalez, I. , Ghorashi, A. , Yadavalli, S. , Lehman, W. J. , Mirzaei, H. , & Douglas, P. M. (2019). Age‐onset phosphorylation of a minor Actin variant promotes intestinal barrier dysfunction. Developmental Cell, 51, 587–601.e7. 31794717 10.1016/j.devcel.2019.11.001 PMC6897307

[acel13742-bib-0030] Fehrenbacher, K. L. , Yang, H.‐C. , Gay, A. C. , Huckaba, T. M. , & Pon, L. A. (2004). Live cell imaging of mitochondrial movement along Actin cables in budding yeast. Current Biology, 14, 1996–2004. 15556861 10.1016/j.cub.2004.11.004

[acel13742-bib-0031] Fisher, K. , Gee, F. , Wang, S. , Xue, F. , Knapp, S. , Philpott, M. , Wells, C. , Rodriguez, M. , Snoek, L. B. , Kammenga, J. , & Poulin, G. B. (2013). Maintenance of muscle myosin levels in adult C. elegans requires both the double bromodomain protein BET‐1 and sumoylation. Biology Open, 2, 1354–1363. 24285704 10.1242/bio.20136007 PMC3863420

[acel13742-bib-0032] Floyd, S. R. , Pacold, M. E. , Huang, Q. , Clarke, S. M. , Lam, F. C. , Cannell, I. G. , Bryson, B. D. , Rameseder, J. , Lee, M. J. , Blake, E. J. , Fydrych, A. , Ho, R. , Greenberger, B. A. , Chen, G. C. , Maffa, A. , Del Rosario, A. M. , Root, D. E. , Carpenter, A. E. , Hahn, W. C. , … Yaffe, M. B. (2013). The bromodomain protein Brd4 insulates chromatin from DNA damage signalling. Nature, 498, 246–250. 23728299 10.1038/nature12147 PMC3683358

[acel13742-bib-0033] Foissner, I. , & Wasteneys, G. O. (2007). Wide‐ranging effects of eight Cytochalasins and Latrunculin a and B on intracellular motility and Actin filament reorganization in Characean Internodal cells. Plant and Cell Physiology, 48, 585–597. 17327257 10.1093/pcp/pcm030

[acel13742-bib-0034] Garcia, G. , Homentcovschi, S. , Kelet, N. , & Higuchi‐Sanabria, R. (2022). Imaging of Actin cytoskeletal integrity during aging in *C. elegans* . Methods in Molecular Biology, 2364, 101–137. 34542850 10.1007/978-1-0716-1661-1_5

[acel13742-bib-0035] Gieseler, K. , Qadota, H. , & Benian, G. M. (2017). Development, structure, and maintenance of *C. elegans* body wall muscle. WormBook, 2017, 1–59. 10.1895/wormbook.1.81.2 PMC5410635 27555356

[acel13742-bib-0036] Greer, E. L. , Becker, B. , Latza, C. , Antebi, A. , & Shi, Y. (2016). Mutation of *C. elegans* demethylase spr5 extends transgenerational longevity. Cell Research, 26, 229–238. 26691751 10.1038/cr.2015.148 PMC4746603

[acel13742-bib-0037] He, Y. , Zan, X. , Miao, J. , Wang, B. , Wu, Y. , Shen, Y. , Chen, X. , Gou, H. , Zheng, S. , Huang, N. , Cheng, Y. , Ju, Y. , Fu, X. , Qian, Z. , Zhou, P. , Liu, J. , & Gao, X. (2022). Enhanced anti‐glioma efficacy of doxorubicin with BRD4 PROTAC degrader using targeted nanoparticles. Materials Today Bio, 16, 100423. 10.1016/j.mtbio.2022.100423 PMC9489811 36157053

[acel13742-bib-0038] Higuchi, R. , Vevea, J. D. , Swayne, T. C. , Chojnowski, R. , Hill, V. , Boldogh, I. R. , & Pon, L. A. (2013). Actin dynamics affect mitochondrial quality control and aging in budding yeast. Current Biology, 23, 2417–2422. 24268413 10.1016/j.cub.2013.10.022 PMC3932488

[acel13742-bib-0039] Higuchi‐Sanabria, R. , Durieux, J. , Kelet, N. , Homentcovschi, S. , de Los Rios Rogers, M. , Monshietehadi, S. , Garcia, G. , Dallarda, S. , Daniele, J. R. , Ramachandran, V. , Sahay, A. , Tronnes, S. U. , Joe, L. , & Dillin, A. (2020). Divergent nodes of non‐autonomous UPRER signaling through serotonergic and dopaminergic neurons. Cell Reports, 33, 108489. 33296657 10.1016/j.celrep.2020.108489 PMC8820220

[acel13742-bib-0040] Higuchi‐Sanabria, R. , Paul Rd, J. W. , Durieux, J. , Benitez, C. , Frankino, P. A. , Tronnes, S. U. , Garcia, G. , Daniele, J. R. , Monshietehadi, S. , & Dillin, A. (2018). Spatial regulation of the Actin cytoskeleton by HSF‐1 during aging. Molecular Biology of the Cell, 29, 2522–2527. 30133343 10.1091/mbc.E18-06-0362 PMC6254583

[acel13742-bib-0041] Higuchi‐Sanabria, R. , Pernice, W. M. A. , Vevea, J. D. , Alessi Wolken, D. M. , Boldogh, I. R. , & Pon, L. A. (2014). Role of asymmetric cell division in life span control in Saccharomyces cerevisiae. FEMS Yeast Research, 14, 1133–1146. 25263578 10.1111/1567-1364.12216 PMC4270926

[acel13742-bib-0042] Holdorf, A. D. , Higgins, D. P. , Hart, A. C. , Boag, P. R. , Pazour, G. J. , Walhout, A. J. M. , & Walker, A. K. (2020). WormCat: An online tool for annotation and visualization of *Caenorhabditis elegans* genome‐scale data. Genetics, 214, 279–294. 31810987 10.1534/genetics.119.302919 PMC7017019

[acel13742-bib-0043] Hsu, A.‐L. , Murphy, C. T. , & Kenyon, C. (2003). Regulation of aging and age‐related disease by DAF16 and heat‐shock factor. Science, 300, 1142–1145. 12750521 10.1126/science.1083701

[acel13742-bib-0044] Huang, M. , Qiu, Q. , Xiao, Y. , Zeng, S. , Zhan, M. , Shi, M. , Zou, Y. , Ye, Y. , Liang, L. , Yang, X. , & Xu, H. (2016). BET Bromodomain suppression inhibits VEGF‐induced angiogenesis and vascular permeability by blocking VEGFR2‐mediated activation of PAK1 and eNOS. Scientific Reports, 6, 23770. 27044328 10.1038/srep23770 PMC4820704

[acel13742-bib-0045] Jang, M. K. , Mochizuki, K. , Zhou, M. , Jeong, H.‐S. , Brady, J. N. , & Ozato, K. (2005). The Bromodomain protein Brd4 is a positive regulatory component of P‐TEFb and stimulates RNA polymerase II‐dependent transcription. Molecular Cell, 19, 523–534. 16109376 10.1016/j.molcel.2005.06.027

[acel13742-bib-0046] Kim, W. , Underwood, R. S. , Greenwald, I. , & Shaye, D. D. (2018). OrthoList 2: A new comparative genomic analysis of human and *Caenorhabditis elegans* genes. Genetics, 210, 445–461. 30120140 10.1534/genetics.118.301307 PMC6216590

[acel13742-bib-0047] Korobova, F. , Ramabhadran, V. , & Higgs, H. N. (2013). An Actin‐dependent step in mitochondrial fission mediated by the ER‐associated formin INF2. Science, 339, 464–467. 23349293 10.1126/science.1228360 PMC3843506

[acel13742-bib-0048] Kuleshov, M. V. , Jones, M. R. , Rouillard, A. D. , Fernandez, N. F. , Duan, Q. , Wang, Z. , Koplev, S. , Jenkins, S. L. , Jagodnik, K. M. , Lachmann, A. , McDermott, M. G. , Monteiro, C. D. , Gundersen, G. W. , & Ma'ayan, A. (2016). Enrichr: A comprehensive gene set enrichment analysis web server 2016 update. Nucleic Acids Research, 44, W90–W97. 27141961 10.1093/nar/gkw377 PMC4987924

[acel13742-bib-0049] Lee, J.‐E. , Park, Y.‐K. , Park, S. , Jang, Y. , Waring, N. , Dey, A. , Ozato, K. , Lai, B. , Peng, W. , & Ge, K. (2017). Brd4 binds to active enhancers to control cell identity gene induction in adipogenesis and myogenesis. Nature Communications, 8, 2217. 10.1038/s41467-017-02403-5 PMC5738375 29263365

[acel13742-bib-0050] Lee, S. S. , Lee, R. Y. N. , Fraser, A. G. , Kamath, R. S. , Ahringer, J. , & Ruvkun, G. (2003). A systematic RNAi screen identifies a critical role for mitochondria in *C. elegans* longevity. Nature Genetics, 33, 40–48. 12447374 10.1038/ng1056

[acel13742-bib-0051] Liang, T. , Zhang, X. , Lai, F. , Lin, J. , Zhou, C. , Xu, X. , Tan, X. , Liu, S. , & Li, L. (2019). A novel bromodomain inhibitor, CPI‐203, serves as an HIV‐1 latency‐reversing agent by activating positive transcription elongation factor b. Biochemical Pharmacology, 164, 237–251. 30991051 10.1016/j.bcp.2019.04.005

[acel13742-bib-0052] Linares‐Saldana, R. , Kim, W. , Bolar, N. A. , Zhang, H. , Koch‐Bojalad, B. A. , Yoon, S. , Shah, P. P. , Karnay, A. , Park, D. S. , Luppino, J. M. , Nguyen, S. C. , Padmanabhan, A. , Smith, C. L. , Poleshko, A. , Wang, Q. , Li, L. , Srivastava, D. , Vahedi, G. , Eom, G. H. , … Jain, R. (2021). BRD4 orchestrates genome folding to promote neural crest differentiation. Nature Genetics, 53, 1480–1492. 34611363 10.1038/s41588-021-00934-8 PMC8500624

[acel13742-bib-0053] Link, C. , & Johnson, C. (2002). Reporter transgenes for study of oxidant stress in *Caenorhabditis elegans* . Methods in Enzymology, 353, 497–505. 12078522 10.1016/s0076-6879(02)53072-x

[acel13742-bib-0054] Love, M. I. , Huber, W. , & Anders, S. (2014). Moderated estimation of fold change and dispersion for RNA‐seq data with DESeq2. Genome Biology, 15, 550. 25516281 10.1186/s13059-014-0550-8 PMC4302049

[acel13742-bib-0055] Lovén, J. , Hoke, H. A. , Lin, C. Y. , Lau, A. , Orlando, D. A. , Vakoc, C. R. , Bradner, J. E. , Lee, T. I. , & Young, R. A. (2013). Selective inhibition of tumor oncogenes by disruption of super‐enhancers. Cell, 153, 320–334. 23582323 10.1016/j.cell.2013.03.036 PMC3760967

[acel13742-bib-0056] Machesky, L. M. , Atkinson, S. J. , Ampe, C. , Vandekerckhove, J. , & Pollard, T. D. (1994). Purification of a cortical complex containing two unconventional actins from Acanthamoeba by affinity chromatography on profilin‐agarose. The Journal of Cell Biology, 127, 107–115. 7929556 10.1083/jcb.127.1.107 PMC2120189

[acel13742-bib-0057] Machesky, L. M. , Mullins, R. D. , Higgs, H. N. , Kaiser, D. A. , Blanchoin, L. , May, R. C. , Hall, M. E. , & Pollard, T. D. (1999). Scar, a WASp‐related protein, activates nucleation of Actin filaments by the Arp2/3 complex. Proceedings of the National Academy of Sciences of the United States of America, 96, 3739–3744. 10097107 10.1073/pnas.96.7.3739 PMC22364

[acel13742-bib-0058] Mary, C. , Scherrer, A. , Huck, L. , Lakkaraju, A. K. K. , Thomas, Y. , Johnson, A. E. , & Strub, K. (2010). Residues in SRP9/14 essential for elongation arrest activity of the signal recognition particle define a positively charged functional domain on one side of the protein. RNA, 16, 969–979. 20348448 10.1261/rna.2040410 PMC2856890

[acel13742-bib-0059] May, J. A. , Ratan, H. , Glenn, J. R. , Lösche, W. , Spangenberg, P. , & Heptinstall, S. (1998). GPIIb‐IIIa antagonists cause rapid disaggregation of platelets pre‐treated with cytochalasin D. evidence that the stability of platelet aggregates depends on normal cytoskeletal assembly. Platelets, 9, 227–232. 16793707 10.1080/09537109876744

[acel13742-bib-0060] McCray, B. A. , & Taylor, J. P. (2008). The role of autophagy in age‐related neurodegeneration. Neurosignals, 16, 75–84. 18097162 10.1159/000109761

[acel13742-bib-0061] Moehle, E. A. , Higuchi‐Sanabria, R. , Tsui, C. K. , Homentcovschi, S. , Tharp, K. M. , Zhang, H. , Chi, H. , Joe, L. , de Los Rios Rogers, M. , Sahay, A. , Kelet, N. , Benitez, C. , Bar‐Ziv, R. , Garcia, G. , Shen, K. , Frankino, P. A. , Schinzel, R. T. , Shalem, O. , & Dillin, A. (2021). Cross‐species screening platforms identify EPS‐8 as a critical link for mitochondrial stress and Actin stabilization. Science Advances, 7, eabj6818. 34714674 10.1126/sciadv.abj6818 PMC8555897

[acel13742-bib-0062] Morley, J. F. , & Morimoto, R. I. (2004). Regulation of longevity in Caenorhabditis elegans by heat shock factor and molecular chaperones. Molecular Biology of the Cell, 15, 657–664. 14668486 10.1091/mbc.E03-07-0532 PMC329286

[acel13742-bib-0063] Moshier, J. A. , Cornell, T. , & Majumdar, A. P. N. (1993). Expression of protease genes in the gastric mucosa during aging. Experimental Gerontology, 28, 249–258. 8344396 10.1016/0531-5565(93)90032-9

[acel13742-bib-0064] Murphy, C. T. , McCarroll, S. A. , Bargmann, C. I. , Fraser, A. , Kamath, R. S. , Ahringer, J. , Li, H. , & Kenyon, C. (2003). Genes that act downstream of DAF‐16 to influence the life span of *Caenorhabditis elegans* . Nature, 424, 277–283. 12845331 10.1038/nature01789

[acel13742-bib-0065] Pathak, S. , Stewart, W. C. L. , Burd, C. E. , Hester, M. E. , & Greenberg, D. A. (2020). Brd2 haploinsufficiency extends life span and healthspan in C57B6/J mice. PLoS One, 15, e0234910. 32559200 10.1371/journal.pone.0234910 PMC7304595

[acel13742-bib-0066] Poteryaev, D. , Squirrell, J. M. , Campbell, J. M. , White, J. G. , & Spang, A. (2005). Involvement of the Actin cytoskeleton and homotypic membrane fusion in ER dynamics in *Caenorhabditis elegans* . Molecular Biology of the Cell, 16, 2139–2153. 15716356 10.1091/mbc.E04-08-0726 PMC1087224

[acel13742-bib-0067] Pruyne, D. , Evangelista, M. , Yang, C. , Bi, E. , Zigmond, S. , Bretscher, A. , & Boone, C. (2002). Role of formins in Actin assembly: Nucleation and barbed‐end association. Science, 297, 612–615. 12052901 10.1126/science.1072309

[acel13742-bib-0068] Qin, X. , Wang, Y. , & Paudel, H. K. (2016). Early growth response 1 (Egr‐1) is a transcriptional activator of β‐secretase 1 (BACE‐1) in the brain*. Journal of Biological Chemistry, 291, 22276–22287. 27576688 10.1074/jbc.M116.738849 PMC5064006

[acel13742-bib-0069] Quinlan, M. E. , Heuser, J. E. , Kerkhoff, E. , & Mullins, R. D. (2005). *Drosophila* spire is an Actin nucleation factor. Nature, 433, 382–388. 15674283 10.1038/nature03241

[acel13742-bib-0070] Reboul, J. , Vaglio, P. , Tzellas, N. , Thierry‐Mieg, N. , Moore, T. , Jackson, C. , Shin‐i, T. , Kohara, Y. , ThierryMieg, D. , Thierry‐Mieg, J. , Lee, H. , Hitti, J. , Doucette‐Stamm, L. , Hartley, J. L. , Temple, G. F. , Brasch, M. A. , Vandenhaute, J. , Lamesch, P. E. , Hill, D. E. , & Vidal, M. (2001). Open‐reading‐frame sequence tags (OSTs) support the existence of at least 17,300 genes in *C. elegans* . Nature Genetics, 27, 332–336. 11242119 10.1038/85913

[acel13742-bib-0071] Sagot, I. , Rodal, A. A. , Moseley, J. , Goode, B. L. , & Pellman, D. (2002). An Actin nucleation mechanism mediated by Bni1 and profilin. Nature Cell Biology, 4, 626–631. 12134165 10.1038/ncb834

[acel13742-bib-0072] Sanson, K. R. , Hanna, R. E. , Hegde, M. , Donovan, K. F. , Strand, C. , Sullender, M. E. , Vaimberg, E. W. , Goodale, A. , Root, D. E. , Piccioni, F. , & Doench, J. G. (2018). Optimized libraries for CRISPRCas9 genetic screens with multiple modalities. Nature Communications, 9, 5416. 10.1038/s41467-018-07901-8 PMC6303322 30575746

[acel13742-bib-0073] Schinzel, R. T. , Higuchi‐Sanabria, R. , Shalem, O. , Moehle, E. A. , Webster, B. M. , Joe, L. , Bar‐Ziv, R. , Frankino, P. A. , Durieux, J. , Pender, C. , Kelet, N. , Kumar, S. S. , Savalia, N. , Chi, H. , Simic, M. , Nguyen, N.‐T. , & Dillin, A. (2019). The hyaluronidase, TMEM2, promotes ER homeostasis and longevity independent of the UPRER. Cell, 179, 1306–1318.e18. 31761535 10.1016/j.cell.2019.10.018 PMC6913896

[acel13742-bib-0074] Shalem, O. , Sanjana, N. E. , Hartenian, E. , Shi, X. , Scott, D. A. , Mikkelson, T. , Heckl, D. , Ebert, B. L. , Root, D. E. , Doench, J. G. , & Zhang, F. (2014). Genome‐scale CRISPR‐Cas9 knockout screening in human cells. Science, 343, 84–87. 24336571 10.1126/science.1247005 PMC4089965

[acel13742-bib-0075] Shen, X. , Ellis, R. E. , Sakaki, K. , & Kaufman, R. J. (2005). Genetic interactions due to constitutive and inducible gene regulation mediated by the unfolded protein response in C. elegans. PLoS Genetics, 1, e37. 16184190 10.1371/journal.pgen.0010037 PMC1231716

[acel13742-bib-0076] Shibata, Y. , Takeshita, H. , Sasakawa, N. , & Sawa, H. (2010). Double bromodomain protein BET‐1 and MYST HATs establish and maintain stable cell fates in *C. elegans* . Development, 137, 1045–1053. 20181741 10.1242/dev.042812

[acel13742-bib-0077] Shin, E.‐Y. , Park, J.‐H. , You, S.‐T. , Lee, C.‐S. , Won, S.‐Y. , Park, J.‐J. , Kim, H.‐B. , Shim, J. , Soung, N.‐K. , Lee, O.‐J. , Schwartz, M. A. , & Kim, E.‐G. (2020). Integrin‐mediated adhesions in regulation of cellular senescence. Science Advances, 6, eaay3909. 32494696 10.1126/sciadv.aay3909 PMC7202880

[acel13742-bib-0078] Sing, C. N. , Garcia, E. J. , Lipkin, T. G. , Huckaba, T. M. , Tsang, C. A. , Coughlin, A. C. , Yang, E. J. , Boldogh, I. R. , Higuchi‐Sanabria, R. , & Pon, L. A. (2021). Identification of a novel modulator of the actin cytoskeleton, mitochondria, nutrient metabolism and life span in yeast. Nature Communications, 3, 2706. 10.1038/s41467-022-30045-9 PMC9110415 35577788

[acel13742-bib-0079] Taylor, R. C. , & Dillin, A. (2013). XBP‐1 is a cell‐nonautonomous regulator of stress resistance and longevity. Cell, 153, 1435–1447. 23791175 10.1016/j.cell.2013.05.042 PMC4771415

[acel13742-bib-0080] Tharp, K. M. , Higuchi‐Sanabria, R. , Timblin, G. A. , Ford, B. , Garzon‐Coral, C. , Schneider, C. , Muncie, J. M. , Stashko, C. , Daniele, J. R. , Moore, A. S. , Frankino, P. A. , Homentcovschi, S. , Manoli, S. S. , Shao, H. , Richards, A. L. , Chen, K.‐H. , Hoeve, J. T. , Ku, G. M. , Hellerstein, M. , … Weaver, V. M. (2021). Adhesionmediated mechanosignaling forces mitohormesis. Cell Metabolism, 33(7), 1322–1341.e13. 34019840 10.1016/j.cmet.2021.04.017 PMC8266765

[acel13742-bib-0081] Thyagarajan, B. , Blaszczak, A. G. , Chandler, K. J. , Watts, J. L. , Johnson, W. E. , & Graves, B. J. (2010). ETS‐4 is a transcriptional regulator of life span in *Caenorhabditis elegans* . PLoS Genetics, 6, e1001125. 20862312 10.1371/journal.pgen.1001125 PMC2940738

[acel13742-bib-0082] Urano, F. , Calfon, M. , Yoneda, T. , Yun, C. , Kiraly, M. , Clark, S. G. , & Ron, D. (2002). A survival pathway for *Caenorhabditis elegans* with a blocked unfolded protein response. The Journal of Cell Biology, 158, 639–646. 12186849 10.1083/jcb.200203086 PMC2174003

[acel13742-bib-0083] Vizioli, M. G. , Liu, T. , Miller, K. N. , Robertson, N. A. , Gilroy, K. , Lagnado, A. B. , Perez‐Garcia, A. , Kiourtis, C. , Dasgupta, N. , Lei, X. , Kruger, P. J. , Nixon, C. , Clark, W. , Jurk, D. , Bird, T. G. , Passos, J. F. , Berger, S. L. , Dou, Z. , & Adams, P. D. (2020). Mitochondria‐to‐nucleus retrograde signaling drives formation of cytoplasmic chromatin and inflammation in senescence. Genes & Development, 34, 428–445. 32001510 10.1101/gad.331272.119 PMC7050483

[acel13742-bib-0084] Wales, P. , Schuberth, C. E. , Aufschnaiter, R. , Fels, J. , García‐Aguilar, I. , Janning, A. , Dlugos, C. P. , Schäfer‐Herte, M. , Klingner, C. , Wälte, M. , Kuhlmann, J. , Menis, E. , Hockaday Kang, L. , Maier, K. C. , Hou, W. , Russo, A. , Higgs, H. N. , Pavenstädt, H. , Vogl, T. , … Wedlich‐Söldner, R. (2016). Calcium‐mediated Actin reset (CaAR) mediates acute cell adaptations. eLife, 5, e19850. 27919320 10.7554/eLife.19850 PMC5140269

[acel13742-bib-0085] Wang, B. , Guo, H. , Yu, H. , Chen, Y. , Xu, H. , & Zhao, G. (2021). The role of the transcription factor EGR1 in cancer. Frontiers in Oncology, 11, 642547. 33842351 10.3389/fonc.2021.642547 PMC8024650

[acel13742-bib-0086] Xie, Z. , Bailey, A. , Kuleshov, M. V. , Clarke, D. J. B. , Evangelista, J. E. , Jenkins, S. L. , Lachmann, A. , Wojciechowicz, M. L. , Kropiwnicki, E. , Jagodnik, K. M. , Jeon, M. , & Ma'ayan, A. (2021). Gene set knowledge discovery with Enrichr. Current Protocols, 1, e90. 33780170 10.1002/cpz1.90 PMC8152575

[acel13742-bib-0087] Xiong, H. , Pears, C. , & Woollard, A. (2017). An enhanced *C. elegans* based platform for toxicity assessment. Scientific Reports, 7, 9839. 28852193 10.1038/s41598-017-10454-3 PMC5575006

[acel13742-bib-0088] Xu, S. , & Chisholm, A. D. (2011). A Gαq‐Ca2+ signaling pathway promotes Actin‐mediated epidermal wound closure in *C. elegans* . Current Biology, 21, 1960–1967. 22100061 10.1016/j.cub.2011.10.050 PMC3237753

[acel13742-bib-0089] Yang, W. , Dierking, K. , & Schulenburg, H. (2016). WormExp: A web‐based application for a Caenorhabditis elegans‐specific gene expression enrichment analysis. Bioinformatics, 32, 943–945. 26559506 10.1093/bioinformatics/btv667

[acel13742-bib-0090] Ye, T. , Feng, J. , Wan, X. , Xie, D. , & Liu, J. (2020). Double agent: *SPDEF* gene with both oncogenic and tumor‐suppressor functions in breast cancer. Cancer Management and Research, 12, 3891–3902. 32547225 10.2147/CMAR.S243748 PMC7259446

[acel13742-bib-0091] Yin, S.‐Q. , Wang, J.‐J. , Zhang, C.‐M. , & Liu, Z.‐P. (2012). The development of MetAP‐2 inhibitors in cancer treatment. Current Medicinal Chemistry, 19, 1021–1035. 22229417 10.2174/092986712799320709

[acel13742-bib-0092] Yoneda, T. , Benedetti, C. , Urano, F. , Clark, S. G. , Harding, H. P. , & Ron, D. (2004). Compartment‐specific perturbation of protein handling activates genes encoding mitochondrial chaperones. Journal of Cell Science, 117, 4055–4066. 15280428 10.1242/jcs.01275

[acel13742-bib-0093] Zarse, K. , Schmeisser, S. , Groth, M. , Priebe, S. , Beuster, G. , Kuhlow, D. , Guthke, R. , Platzer, M. , Kahn, C. R. , & Ristow, M. (2012). Impaired insulin/IGF1 signaling extends life span by promoting mitochondrial L‐proline catabolism to induce a transient ROS signal. Cell Metabolism, 15, 451–465. 22482728 10.1016/j.cmet.2012.02.013 PMC4844853

[acel13742-bib-0094] Zhang, H. , Li, G. , Zhang, Y. , Shi, J. , Yan, B. , Tang, H. , Chen, S. , Zhang, J. , Wen, P. , Wang, Z. , Pang, C. , Li, J. , Guo, W. , & Zhang, S. (2019). Targeting BET proteins with a PROTAC molecule elicits potent anticancer activity in HCC cells. Frontiers in Oncology, 9, 1471. 31993368 10.3389/fonc.2019.01471 PMC6971110

