# Supplementary material for: Large‐scale genetic screens identify BET‐1 as a cytoskeleton regulator promoting actin function and life span
Source: Aging Cell. 2022 Nov 20;22(1):e13742. doi: 10.1111/acel.13742 (PMC9835578; doi:10.1111/acel.13742)
Supplement: Supplementary file 1 — Figures S1–S9 [file ACEL-22-e13742-s009.pdf]

SUPPLEMENTARY FIGURES, TABLES, LEGENDS.

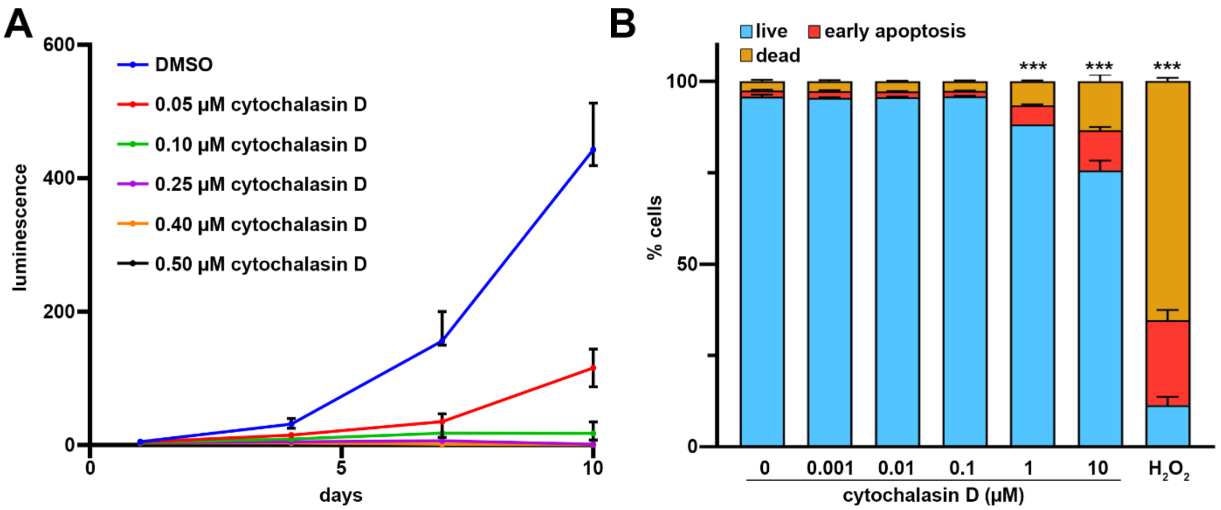

Fig. S1. Low concentrations of cytochalasin D affects growth rate without inducing apoptosis. (A) Cell density was determined via CellTiter-Glo analysis in BJ fibroblasts at day 1, day 4, and day 10 of exposure to indicated concentration of cytochalasin D or DMSO control as described in **Materials and Methods**. n = 4 per condition and data is represented as median +/- interquartile range. **(B)** BJ fibroblasts were treated with cytochalasin D at indicated doses for 24 hours or 30 mM H_2O_2 for 3 hours as a positive control for inducing cell death. Cell death was measured using annexin V and Live/Dead staining followed by flow cytometry as described in **Materials and Methods**. Two-way ANNOVA with Dunnetts test was used to compare the ratio of live cells between control (0) and each drug-treated group. n = 3 with 2 biological replicates.

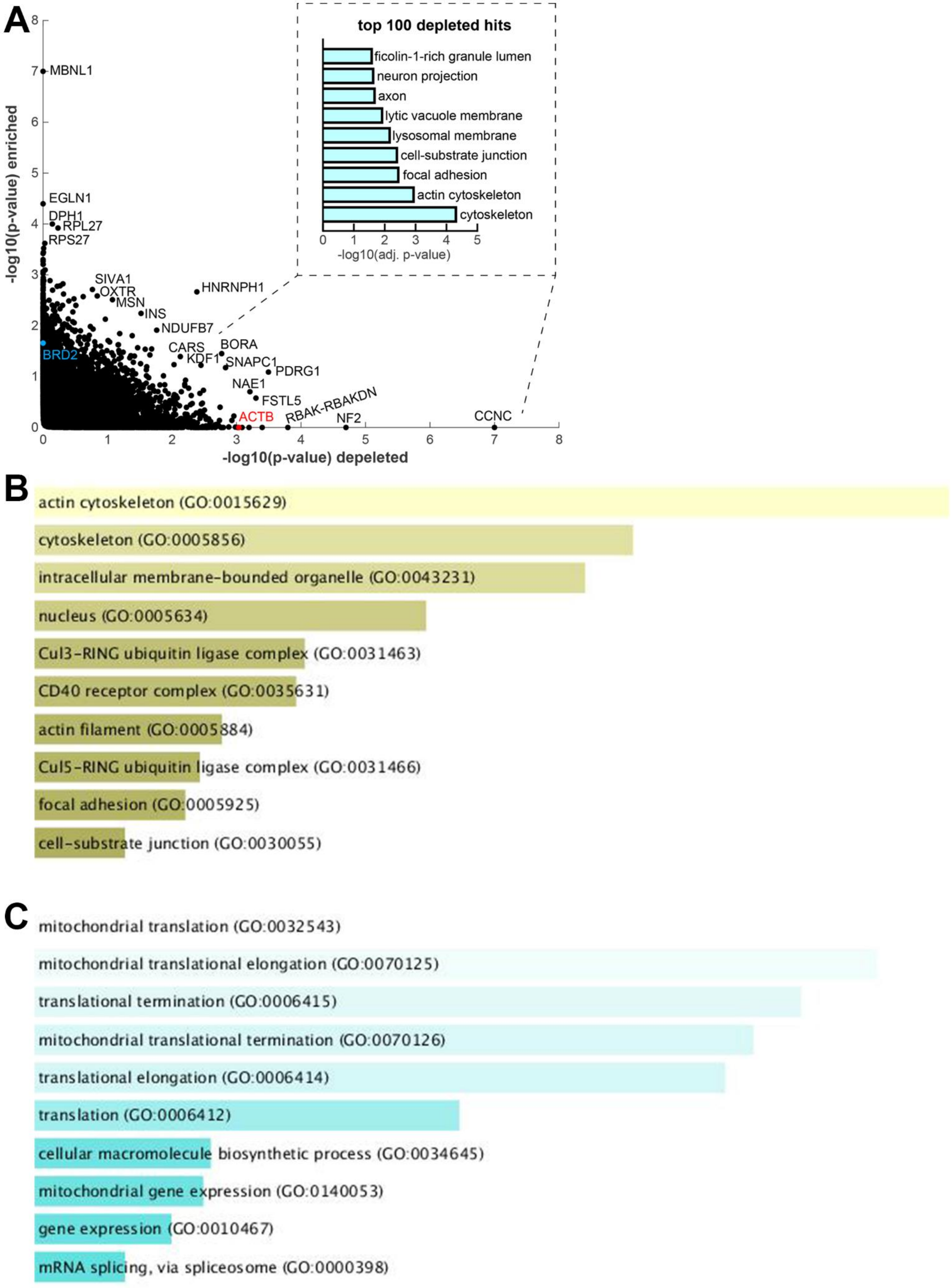
A
top 100 depleted hits
ficolin-1-rich granule lumen
neuron projection
axon
lytic vacuole membrane
lysosomal membrane
cell-substrate junction
focal adhesion
actin cytoskeleton
cytoskeleton
-log10(adj. p-value)
-log10(p-value) enriched
-log10(p-value) depeleted
MBNL1
EGLN1
DPH1
RPL27
RPS27
SIVA1
OXTR
MSN
INS
NDUFB7
HNRNPH1
BRD2
CARS
KDF1
BORA
SNAPC1
PDRG1
NAE1
FSTL5
ACTB
RBAK-RBAKDN
NF2
CCNC
B
actin cytoskeleton (GO:0015629)
cytoskeleton (GO:0005856)
intracellular membrane-bounded organelle (GO:0043231)
nucleus (GO:0005634)
Cul3-RING ubiquitin ligase complex (GO:0031463)
CD40 receptor complex (GO:0035631)
actin filament (GO:0005884)
Cul5-RING ubiquitin ligase complex (GO:0031466)
focal adhesion (GO:0005925)
cell-substrate junction (GO:0030055)
C
mitochondrial translation (GO:0032543)
mitochondrial translational elongation (GO:0070125)
translational termination (GO:0006415)
mitochondrial translational termination (GO:0070126)
translational elongation (GO:0006414)
translation (GO:0006412)
cellular macromolecule biosynthetic process (GO:0034645)
mitochondrial gene expression (GO:0140053)
gene expression (GO:0010467)
mRNA splicing, via spliceosome (GO:0000398)

Fig. S2. Cytochalasin CRISPR-Cas9 screening. (A) The p-values (max p-val) of each gene when calculated for enriched and depleted genes, upon cytochalasin D treatment, is shown. (inset) enrichment analysis for cellular components of the top 100 depleted genes (Chen et al., 2013). All significantly depleted **(B)** and enriched **(C)** genes were defined as those with a p-value < 0.05 and can be found in **Table S1-2**. Complete lists of GO terms are found in **Table S3** (depleted) and **Table S4** (enriched). Gene lists were run through Enrichr and gene ontologies (GO) for cellular component were graphed and sorted by p-value ranking. All raw counts are available on **Table S5**.

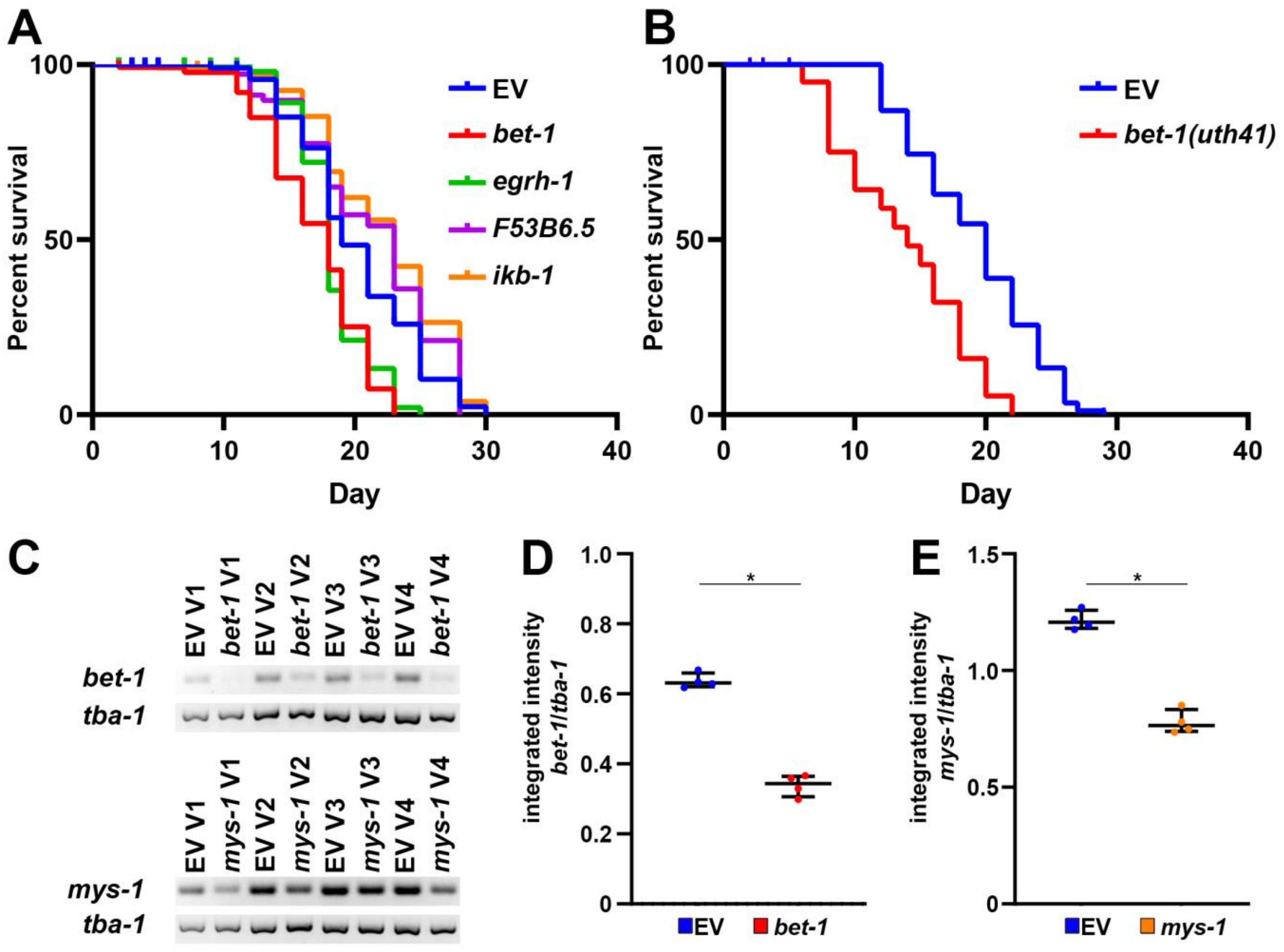

Fig. S3. Knockdown and knockout of *bet-1* decrease lifespan. (A) Lifespans of wild-type, N2 animals grown on empty vector (EV, blue, p-values against N2), *bet-1* (red, $p < 0.001$), *egrh-1* (green, $p < 0.001$), *F53B6.5* (purple, p = not significant), or *ikb-1* (orange, p = not significant) RNAi from hatch. See **Table S6-7** for complete lifespan statistics. **(B)** Lifespans of wild-type N2 animals (blue) and *bet-1(uth41)* mutant animals (red) grown on empty vector RNAi from hatch. $p < 0.001$. See **Table S6-7** for complete lifespan statistics. **(C)** RT-PCR of transcripts in N2 animals grown on empty vector (EV), *bet-1*, or *mys-1* RNAi from hatch. RNA was isolated in day 1 adults, followed by cDNA synthesis and PCR. Quantification was performed in ImageJ measuring the integrated intensity of bands and normalizing against a *tba-1* loading control. **B** Image shows PCR band for each of four biological replicates (V1-V4). Right side are dot plots for *bet-1* RNAi **(D)** and *mys-1* RNAi **(E)** where each dot represents a single biological replicate and lines represent median and interquartile range. * = $p < 0.05$ calculated using non-parametric Mann-Whitney testing.

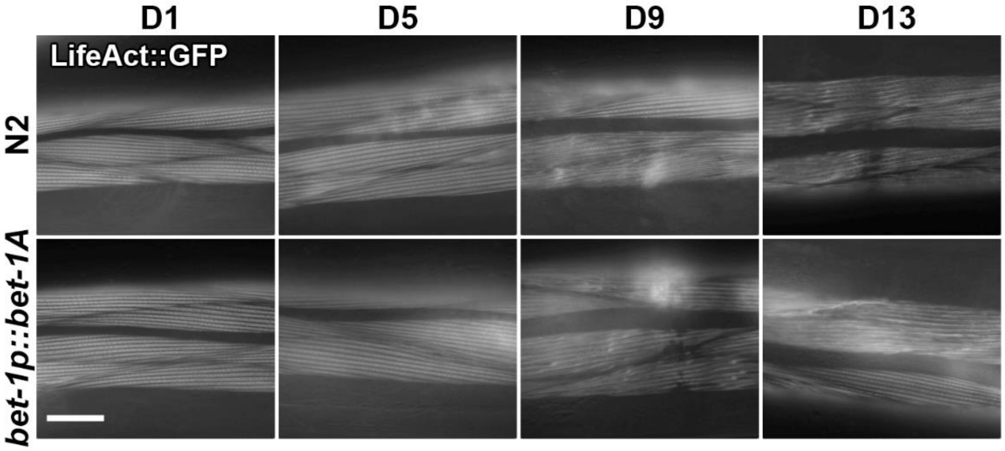

Fig. S4. *bet-1A* overexpression does not impact actin filaments during aging. Representative fluorescent images of adult animals expressing LifeAct::GFP from a muscle-specific promoter, *myo-3p*. Wild-type, N2 and *bet-1A* overexpression (*bet-1p::bet-1A*) animals were grown on empty vector (EV) RNAi from hatch and imaged at day 1, 5, 9, and 13 of adulthood. Images were captured on a Leica Thunder Imager. Scale bar is 10 µm.

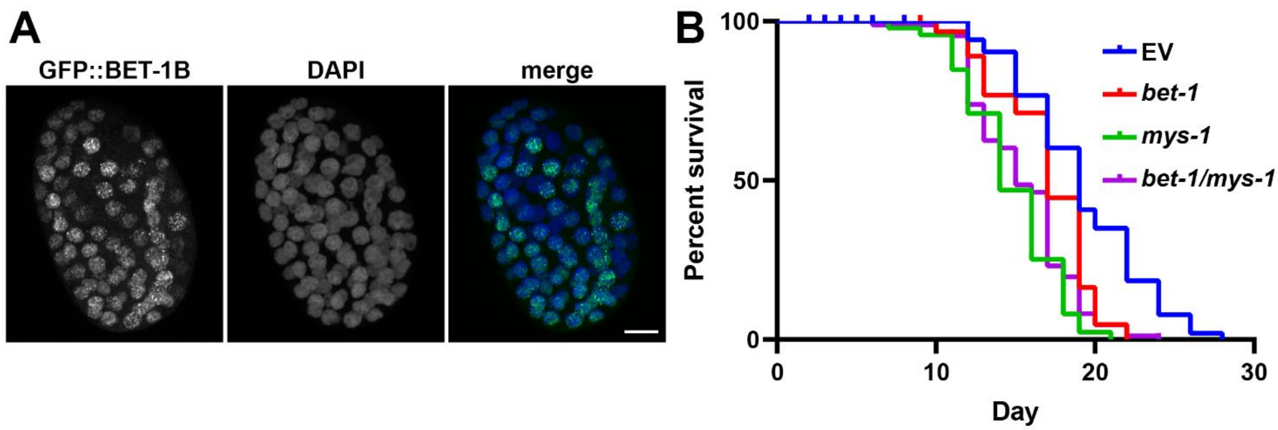

Fig. S5. BET-1 functions as a transcriptional regulator. (A) Animals overexpressing *GFP::bet-1B* were grown on empty vector RNAi from hatch. Eggs were isolated by a standard bleaching protocol, fixed using 4% PFA, and counterstained with DAPI as described in STAR Methods. Images were collected on a Leica Stellaris 5 confocal. Scale bar is 5 µm. **(B)** Lifespans of wild-type, N2 animals grown on empty vector (EV, blue) or a 50/50 mix of EV/*bet-1* (red), EV/*mys-1* (green), or *bet-1/mys-1* (purple) from hatch. See **Table S6-7** for lifespan statistics. All p-values < 0.0001 vs. N2.

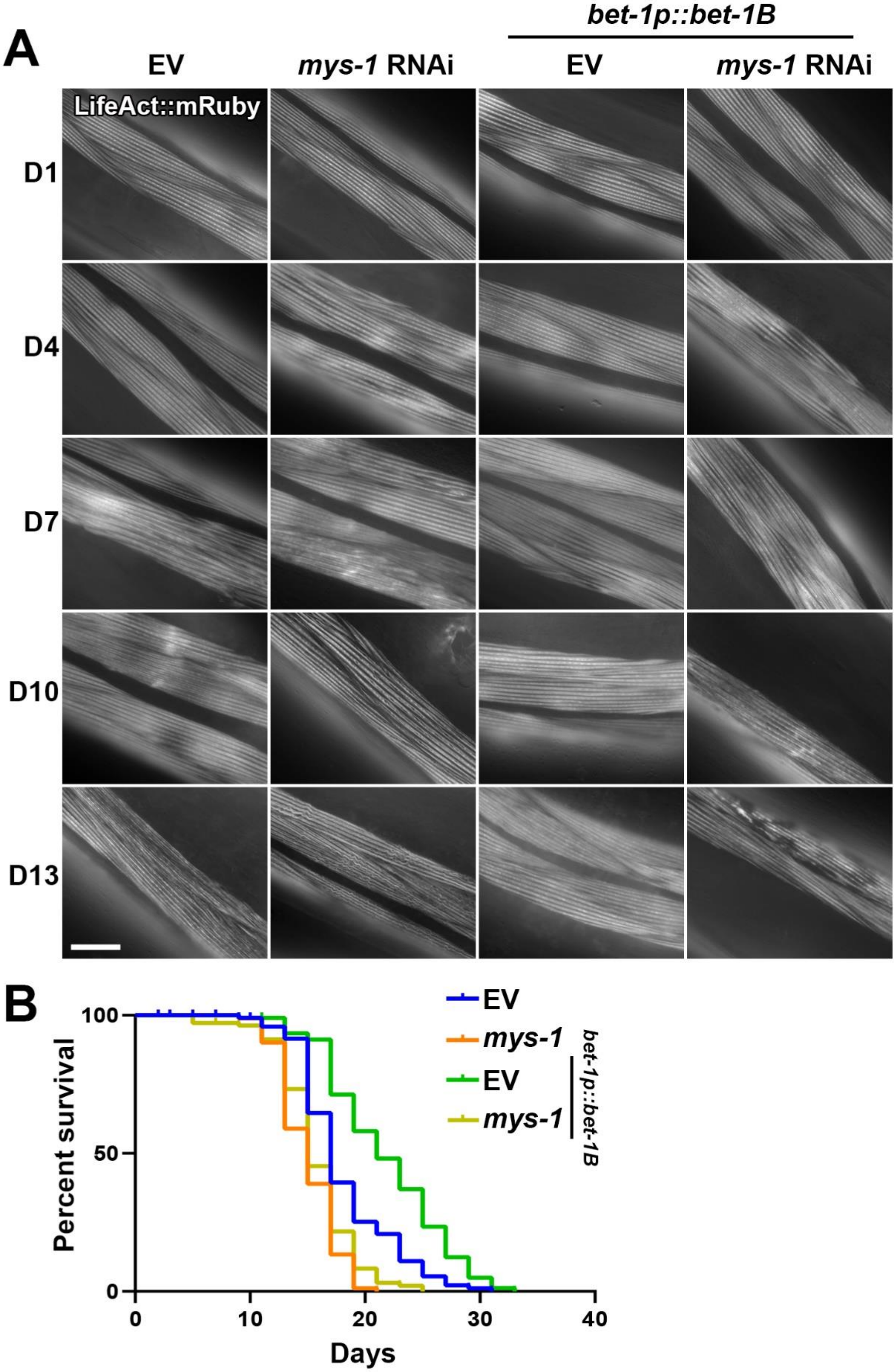

A
bet-1p::bet-1B
EV
mys-1 RNAi
EV
mys-1 RNAi
LifeAct::mRuby
D1
D4
D7
D10
D13
B
Percent survival
100
50
0
0
10
20
30
40
Days
EV
mys-1
EV
mys-1
bet-1p::bet-1B

Fig. S6. *mys-1* is required for BET-1 mediated effects on actin and lifespan. (A) Representative fluorescent images of adult animals expressing LifeAct::mRuby from a muscle-specific promoter, *myo-3p*. Wild-type, N2 and *bet-1B* overexpression (*bet-1p::bet-1B*) animals were grown on empty vector (EV) or *mys-1* RNAi from hatch and imaged at day 1, 4, 7, 10, and 13 of adulthood. Images were captured on a Zeiss AxioObserver.Z1. Scale bar is 10 µm. **(B)** Lifespans of N2 (EV, blue) and *bet-1B* overexpression (*bet-1p::bet-1B*, green) animals grown on EV or *mys-1* RNAi (N2, orange; *bet-1B* overexpression, yellow) from hatch. N2, EV (blue) vs. *bet-1p::bet-1B*, EV (green) = p <0.0001; N2, *mys-1* RNAi (orange) vs. *bet-1p::bet-1, mys-1* RNAi (yellow) = not significant. See **Table S6-7** for complete lifespan statistics.

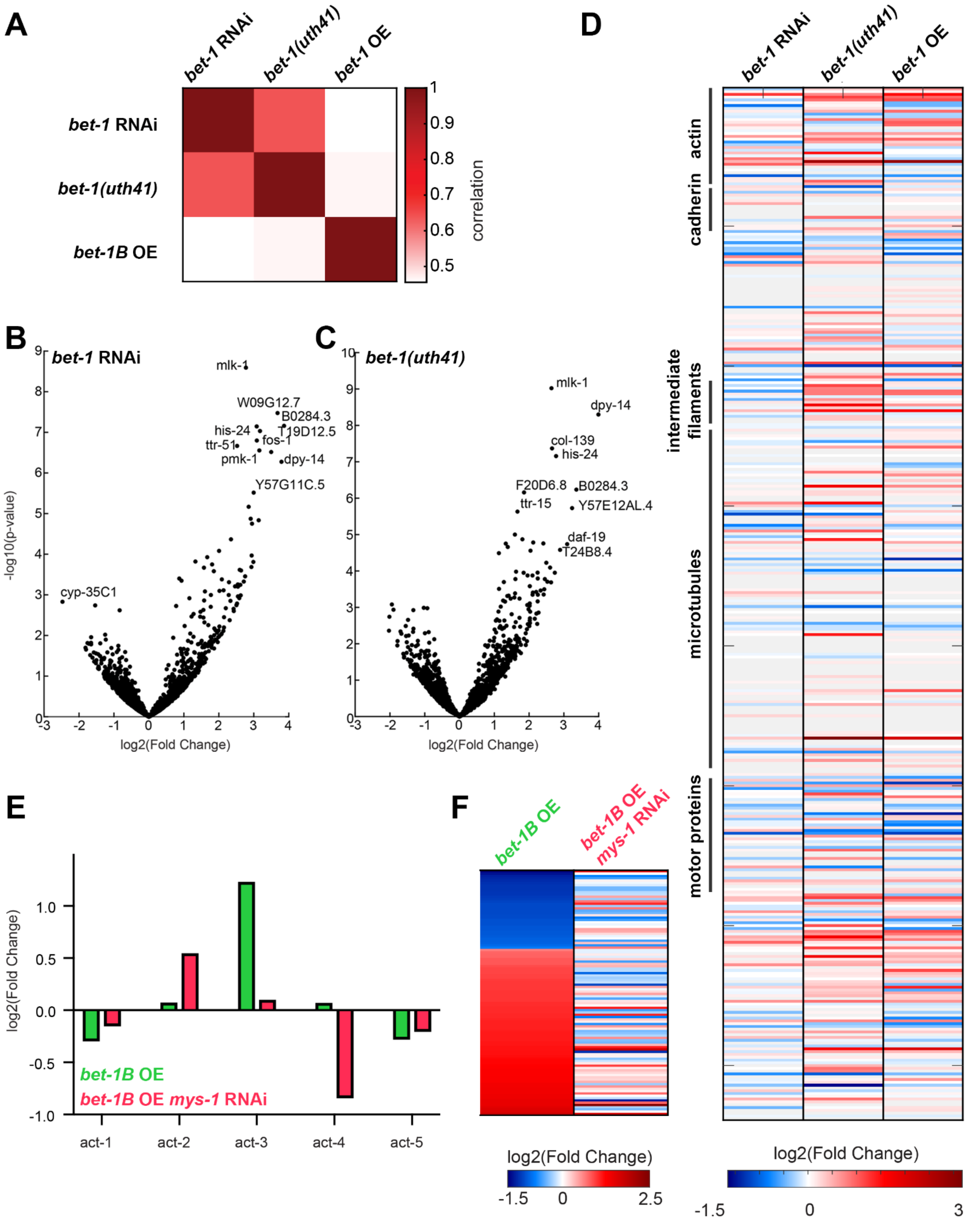
A
bet-1 RNAi
bet-1(uth41)
bet-1 OE
bet-1 RNAi
bet-1(uth41)
bet-1B OE
correlation
1
0.9
0.8
0.7
0.6
0.5
B
bet-1 RNAi
mlk-1
W09G12.7
B0284.3
his-24
T19D12.5
ttr-51
fos-1
pmk-1
dpy-14
Y57G11C.5
cyp-35C1
-log10(p-value)
log2(Fold Change)
C
bet-1(uth41)
mlk-1
dpy-14
col-139
his-24
F20D6.8
B0284.3
ttr-15
Y57E12AL.4
daf-19
T24B8.4
log2(Fold Change)
D
bet-1 RNAi
bet-1(uth41)
bet-1 OE
actin
cadherin
intermediate filaments
microtubules
motor proteins
log2(Fold Change)
-1.5
0
3
E
log2(Fold Change)
bet-1B OE
bet-1B OE mys-1 RNAi
act-1
act-2
act-3
act-4
act-5
F
bet-1B OE
bet-1B OE mys-1 RNAi
log2(Fold Change)
-1.5
0
2.5

Fig. S7. Gene expression analysis of bet-1 knockdown, knockout, and over-expression. **(A)** Pearson's correlation of log2(fold changes) in gene expression between the different datasets, normalized by N2 wild-type control. Gene expression changes, as in Figure 3A, for *bet-1* RNAi **(B)** or *bet-1* CRISPR mutant **(C)**. **(D)** Gene expression changes for all cytoskeleton genes as annotated in WormCat (Holdorf et al., 2020): actin function; cadherin; centrosome; claudin; innexin; integrin; intermediate filament protein; microtubule; motor protein; other. **(E-F)** Changes in differentially expressed genes is dependent on *mys-1*: Gene expression of *bet-1B* over-expressing worms subjected to *mys-1* RNAi was compared to a *mys-1* RNAi only baseline control. The five actin genes are plotted in **(E)**, and all differentially expressed genes in the *bet-1B* over-expressing worms are plotted in **(F)**. See **Table S8** for all differentially expressed genes.

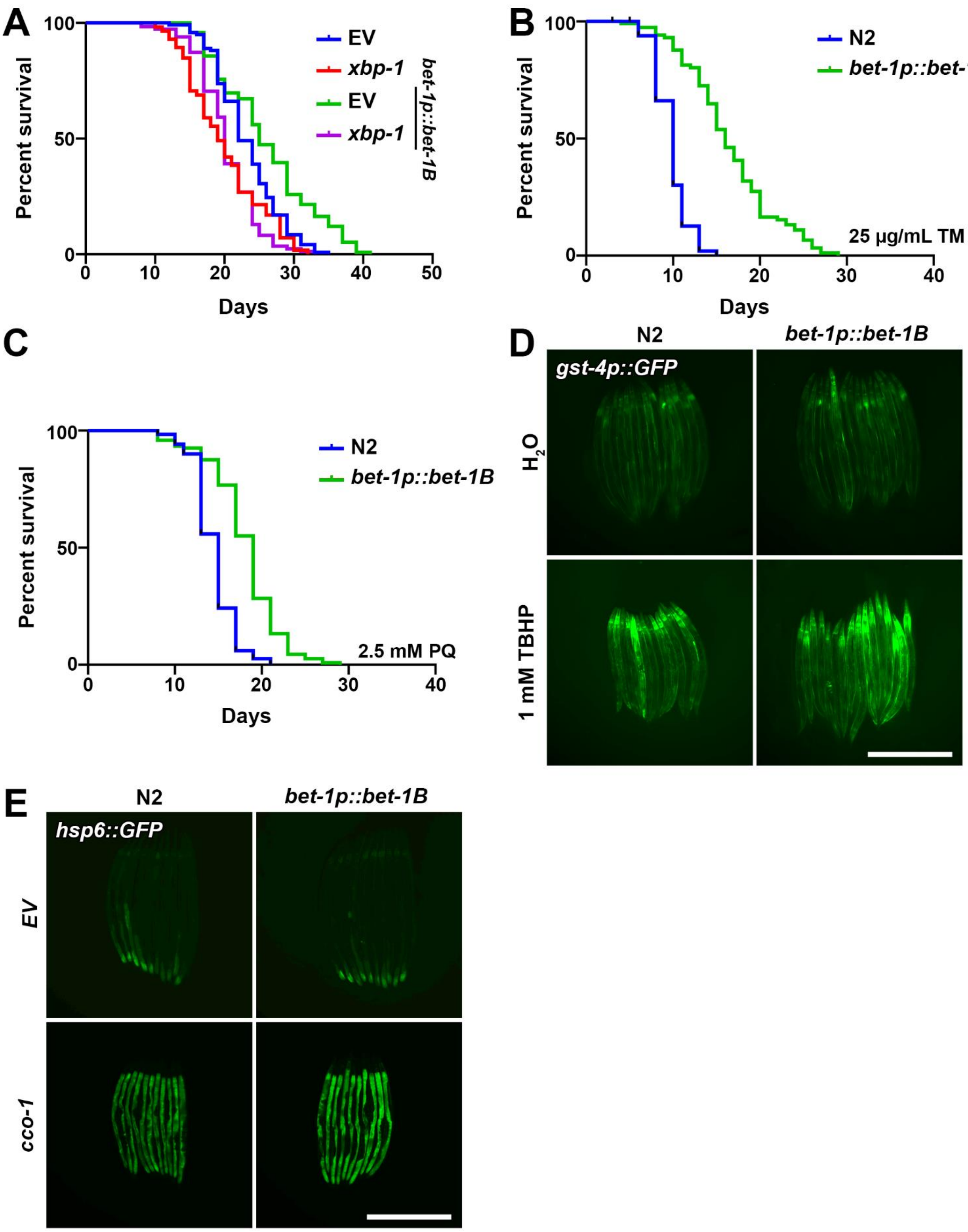
A
Percent survival
Days
EV
xbp-1
EV
xbp-1
bet-1p::bet-1B
B
N2
bet-1p::bet-1B
25 µg/mL TM
C
2.5 mM PQ
D
N2
bet-1p::bet-1B
gst-4p::GFP
H2O
1 mM TBHP
E
hsp6::GFP
EV
cco-1

Fig. S8. *bet-1B* overexpression promotes ER and mitochondrial stress resilience. (A) Lifespans of N2 (EV, blue) and *bet-1B* overexpression (*bet-1p::bet-1B*, green) animals grown on EV or *xbp-1* RNAi (N2, red; *bet-1B* overexpression, purple) from hatch. N2, EV (blue) vs. *bet-1p::bet-1B*, EV (green) = p <0.0001; N2, *xbp-1* RNAi (red) vs. *bet-1p::bet-1, xbp-1* RNAi (purple) = not significant. See **Table S6-7** for complete lifespan statistics. Fig. S8A, S9A, and S9B were performed simultaneously and can be directly compared. **(B)** Survival curve of N2 (blue) and *bet-1B* overexpression (*bet-1p::bet-1B*, green) animals grown on EV from hatch and moved onto plates containing 25 µg/mL tunicamycin on day 1 of adulthood. p < 0.0001. See **Table S6-7** for complete lifespan statistics. **(C)** Survival curve of N2 (blue) and *bet-1B* overexpression (*bet-1p::bet-1B*, green) animals grown on EV from hatch and moved onto plates containing 2.5 mM paraquat on L4. p < 0.0001. See **Table S6-7** for complete lifespan statistics. **(D)** Representative fluorescent images of adult animals expressing *gst-4p::GFP* as a marker for oxidative stress response (SKN-1 transcriptional reporter). Animals were treated with water or 1 mM tert-butyl hydroperoxide (TBHP) as described in **Materials and Methods**. Scale bar is 500 µm. **(E)** Representative fluorescent images of adult animals expressing *hsp-6p::GFP* as a marker for the unfolded protein response of the mitochondria (UPR^{MT}). Animals were grown on EV or *cco-1* RNAi (mitochondrial stress by inhibition of electron transport chain) from hatch and imaged at day 1 of adulthood. Scale bar is 500 µm.

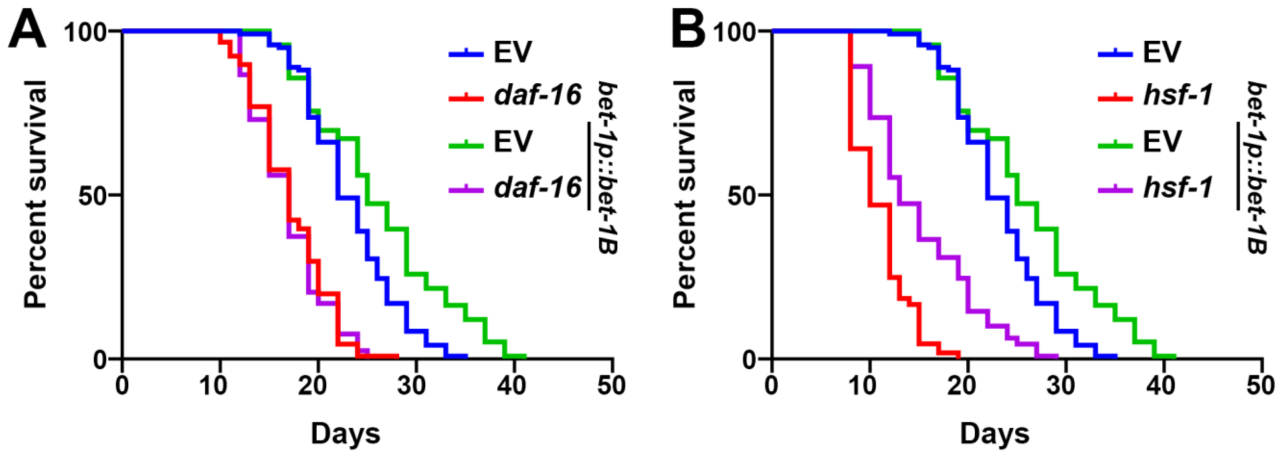

Fig. S9. Longevity of *bet-1B* overexpressing animals is dependent on *daf-16* and not *hsf-1*. (A) Lifespans of N2 (EV, blue) and *bet-1B* overexpression (*bet-1p::bet-1B*, green) animals grown on EV or *daf-16* RNAi (N2, red; *bet-1B* overexpression, purple) from hatch. N2, EV (blue) vs. *bet-1p::bet-1B*, EV (green) = p <0.0001; N2, *daf-16* RNAi (red) vs. *bet-1p::bet-1, daf-16* RNAi (purple) = not significant. See **Table S6-7** for complete lifespan statistics. **(B)** Lifespans of N2 (EV, blue) and *bet-1B* overexpression (*bet-1p::bet-1B*, green) animals grown on EV or *hsf-1* RNAi (N2, red; *bet-1B* overexpression, purple) from hatch. N2, EV (blue) vs. *bet-1p::bet-1B*, EV (green) = p <0.0001; N2, *hsf-1* RNAi (red) vs. *bet-1p::bet-1, hsf-1* RNAi (purple) = p < 0.0001. See **Table S6-7** for complete lifespan statistics. Fig. S8A, S9A, and S9B were performed simultaneously and can be directly compared.

Table S1. Enriched genes from cytochalasin screen.

Table S2. Depleted genes from cytochalasin screen.

Table S3. GO Terms for depleted genes from cytochalasin screen.

Table S4. GO Terms for enriched genes from cytochalasin screen.

Table S5. Raw sgRNA counts from cytochalasin screen.

Table S6. Statistics for lifespans.

Table S7. Replicates for lifespans.

Table S8. Differentially expressed genes from RNA-seq.
